# Isoindolinones as Michael Donors under Phase Transfer Catalysis: Enantioselective Synthesis of Phthalimidines Containing a Tetrasubstituted Carbon Stereocenter

**DOI:** 10.3390/molecules20058484

**Published:** 2015-05-12

**Authors:** Francesco Scorzelli, Antonia Di Mola, Laura Palombi, Antonio Massa

**Affiliations:** Dipartimento di Chimica e Biologia, Università di Salerno, Via Giovanni Paolo II, 132, 84084-Fisciano, SA, Italy; E-Mails: scorzelli.francesco@tiscali.it (F.S.); toniadimola@libero.it (A.D.M.); lpalombi@unisa.it (L.P.)

**Keywords:** synthetic methods, asymmetric catalysis, nitrogen heterocycles, conjugate addition, chiral phase transfer catalysts

## Abstract

Readily available chiral ammonium salts derived from cinchona alkaloids have proven to be effective phase transfer catalysts in the asymmetric Michael reaction of 3-substituted isoindolinones. This protocol provides a convenient method for the construction of valuable asymmetric 3,3-disubstituted isoindolinones in high yields and  moderate to good enantioselectivity. Diastereoselectivity was also investigated in the construction of contiguous tertiary and quaternary stereocenters. The use of acrolein as Michael acceptor led to an interesting tricyclic derivative, a pyrroloisoindolinone analogue, via a tandem conjugated addition/cyclization reaction.

## 1. Introduction

**Figure 1:**
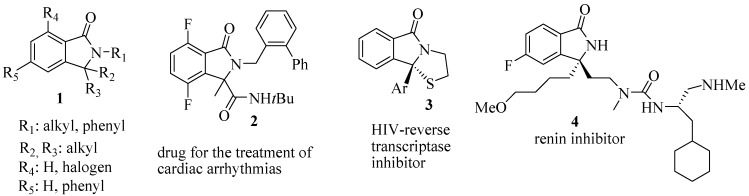
Examples of synthetic pharmacalogically active chiral 3,3-disubstituted isoindolinones.

The construction of chiral tetrasubstituted carbons represents one of the most challenging and demanding topics in the synthesis of natural products and chiral drugs [1,2,3,4,5]. The development of such a new catalytic enantioselective synthesis of isoindolinones with this feature appeared to be of great value. Besides unsubstituted [6,7] and monosubstituted isoindolinones [7], many asymmetric 3,3-disubstituted isoindolinones show a wide spectra of biological activities as represented by the general structure **1**, a family of inhibitors of phosphatidylinositolo 3-kinase [8]; by **2**, a drug for the treatment of cardiac arrhythmias [9]; by **3**, which is a HIV-reverse transcriptase inhibitor [10]; and by **4**, which is a renin inhibitor [11] (Figure 1). 

**Figure 2:**
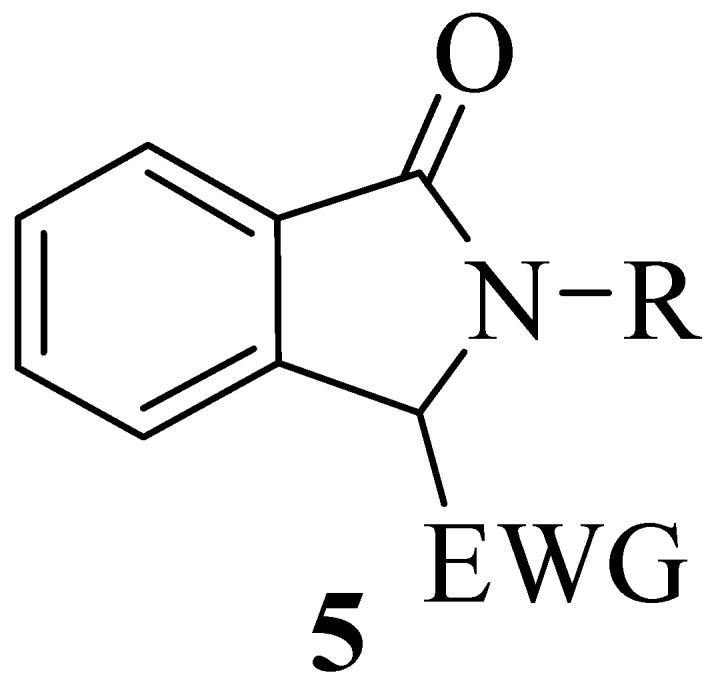
General structure of potentially nucleophilic 3-substituted isoindolinones

The preparation of these compounds in enantioenriched form has traditionally been pursued by kinetic resolution of racemates or with chiral acids or bases [12,13,14] or by the use chiral auxiliaries [15,16,17], while few catalytic asymmetric methodologies have been reported [18,19,20,21,22,23,24,25]. In particular, the construction of tetrasubstituted stereocenters in the heterocyclic ring in the presence of a chiral Pd(II) complex in an aerobic aza-Wacker-type cyclization performed on alkylidene *ortho-*substituted benzamides has been reported in 2012 by Zhang *et al.* [19]. In 2013 Nishimura *et al.* found that a chiral hydroxorhodium complex was effective in the synthesis of 3,3-diaryl substituted isoindolinones [20]. Only one organocatalytic method for the asymmetric Friedel–Crafts alkylation of indoles with 3-alkyl-3-hydroxyisoindolin-1-ones, showing good enantioselectivity has been described by Zhou *et al.* in 2011 [21]. The limited number of catalytic methodologies for the construction of quaternary stereocenters on the isoindolinone ring prompted us to tackle this challenge, considering the possible use of compounds of general structure **5** as nucleophiles in asymmetric reactions (Figure 2). 

The electron-withdrawing group should activate the benzylic carbon in the 3 position of **5** for asymmetric transformations in the presence of chiral organocatalysts or chiral phase transfer catalysts. As part of our ongoing studies on the asymmetric synthesis of isoindolinones and related compounds [24,25,26,27,28], we report herein the first example of an enantioselective Michael reaction of *rac*-3-substituted isoindolinones in the presence of chiral phase transfer catalysts for the construction of 3,3-disubstituted chiral derivatives, highlighting the scope and the limitations of the procedure.

## 2. Results and Discussion

**Figure 3:**
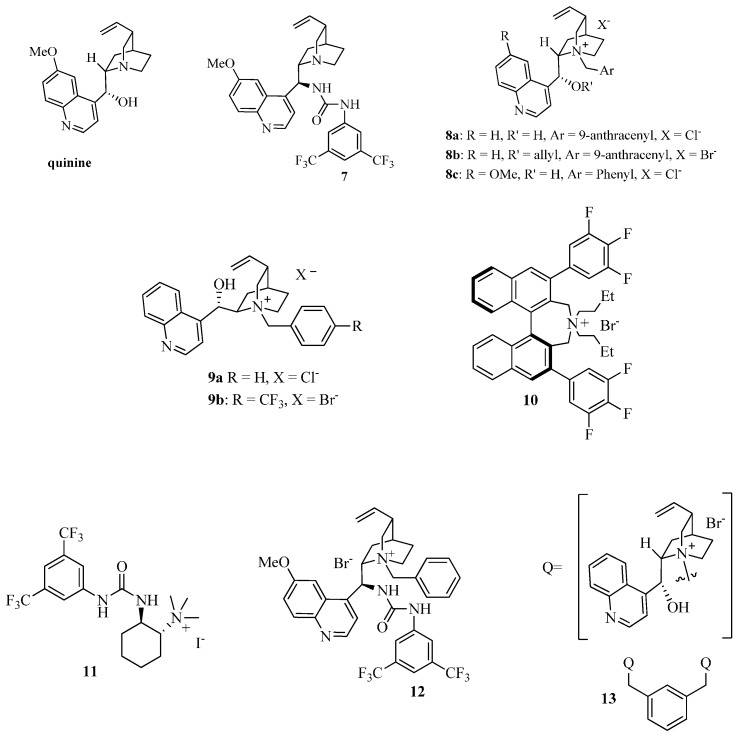
A survey of chiral phase transfer catalysts and organocatalysts.

In order to prove the synthetic utility of compounds of general structure **5** in asymmetric transformations, we started our investigation by testing the reactivity of the readily available isoindolinone **5a** [29] taken as model compound. The choice to study the asymmetric Michael reaction has been inspired by the number of asymmetric methodologies using cyclic β-keto esters and activated phthalides, which can be performed under both organocatalytic [30,31,32] or chiral phase transfer conditions [33,34,35]. Accordingly, we firstly tested quinine and bifunctional organocatalyst **7** (Figure 3), under the conditions of Table 1. Pleasingly, the reaction of **5a** with methyl vinyl ketone in DCM led to the adduct **6a** in moderate ee. However, rather low yields, very long reaction times and incomplete conversions were observed (Table 1). 

**Table 1:** Chinchona based organocatalysts in the and identified Michael reaction of 3-substituted isoindolinones. 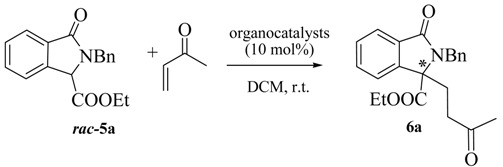

Entry	Cat. (10 mol %)	t (h)	Yield (%) ^a^	ee (%) ^b^
1	**quinine**	96	51	48
2	**7**	96	55	45

^a^ Isolated yield. ^b^ Determined by HPLC on chiral column.

Then, for comparison, we turned our attention to the use of the chiral phase transfer catalyst **8a** (Figure 3) in combination with the inorganic base K_2_CO_3_, a catalytic system also employed in Michael reaction of cyclic β-keto esters with good results [33,34,35]. Nicely enough, the expected Michael adduct **6a** was obtained in high yield and with higher enantioselectivity (56% ee) than when quinine and **7** were used and in a shorter reaction time (compare Entry 1 of Table 2 with the data of Table 1). Considering the promising results obtained under asymmetric phase transfer conditions, we tested other readily available chiral ammonium salts, widely used in asymmetric reactions [33,34,35,36,37,38,39]. The *O*-allyl ether derivative **8b** was less effective in terms of yield and enantioselectivity, emphasizing the importance of maintaining free the -OH group at the C-9 position of the catalyst (Entry 2). The *quasi*-enantiomer cinchoninium salt **9a** showed a comparable efficiency with respect to **8a**, giving *ent*-**6a** with a −55% ee (Entry 3), while **8c** had a negative effect on the enantioselectivity (entry 4).

**Table 2:** Phase transfer catalyzed asymmetric Michael reactions. 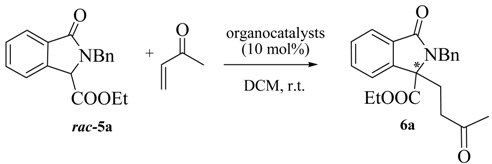

Entry	PTC (10 mol %)	Solvent	T (°C)	t (h)	Yield (%) ^a^	ee (%) ^b^
1	**8a**	CH_2_Cl_2_	r.t.	2	90	56
2	**8b**	CH_2_Cl_2_	r.t.	24	95	11
3	**9a**	CH_2_Cl_2_	r.t.	2	89	−55
4	**8c**	CH_2_Cl_2_	r.t.	48	92	38
5	**10**	CH_2_Cl_2_	r.t.	30	91	10
6	**11**	CH_2_Cl_2_	r.t.	8	83	rac.
7	**12**	CH_2_Cl_2_	r.t.	8	96	−11
8 ^c^	**8a**	CH_2_Cl_2_	r.t.	3	96	56
9 ^d^	**8a**	CH_2_Cl_2_	r.t.	24	93	54
10	**8a**	CH_2_Cl_2_/H_2_O	r.t.	1	92	40
11	**8a**	CH_2_Cl_2_	−20	3	91	60
12	**8a**	CH_2_Cl_2_	−40	24	97	73
13	**8a**	CH_2_Cl_2_	−50	48	97	68
14	**9b**	CH_2_Cl_2_	−50	48	97	−25
15 ^e^	**8a**	CH_2_Cl_2_	−40	48	97	65
16 ^f^	**8a**	CH_2_Cl_2_	−40	48	62	33
17	**8a**	CHCl_3_	−40	72	96	68
18	**8a**	1,2-DCE	r.t.	24	97	51
19	**8a**	Toluene	−40	72	87	61
20 ^g^	**8a**	CH_2_Cl_2_	−40	36	95	68
21 ^h^	**8a**	CH_2_Cl_2_	−40	7	94	63

^a^ Isolated yield. ^b^ Determined by HPLC on chiral column. ^c^ **8a** was used at 5 mol %. ^d^ **8a** was used at 2 mol %. ^e^ Cs_2_CO_3_ was used. ^f^ *i*Pr_2_NEt was used. ^g^ [**5a**] = 7 mM instead of 14 mM of entry 12. ^h^ [**5a**] = 28 mM.

The structurally different Maruoka’s catalyst **10** [33] also employed in a number of asymmetric transformations, gave almost a racemic compound (entry 5). We also investigated the bifunctional chiral ammonium salts **11** [36,37] and **12** [38] derived from (*R,R*)-diamino cyclohexane and from quinine, respectively. Despite the possibility of giving a more ordered TS with the additional hydrogen bonds of the urea group [39], unsatisfactory results were obtained (Entries 6 and 7). Thus, focusing on **8a**, we were able to perform the reaction even at 2 mol % with only a slight decrease in the ee (Entries 8 and 9). The DCM/H_2_O system was less effective in terms of enantioselectivity (entry 10). Only with the decreasing of the temperature we observed an increase of the enantioselectivity with a maximum of 73% ee at −40 °C (Entries 11–13). Under these conditions, the PTC **9b** was less effective (entry 14). Other combinations of bases like Cs_2_CO_3_ or *i*Pr_2_NEt with DCM and solvents like CHCl_3_, 1,2-DCE or toluene with K_2_CO_3_, gave less satisfactory results, even if in some cases they have positive effects on asymmetric Michael reactions of methyl vinyl ketone (Entries 15–19) [34]. Also the effect of the molar concentration was analyzed: the best result is represented by Entry 12 in comparison with those of Entries 20 and 21. Then, the scope of the reaction was analyzed by screening several Michael acceptors and isoindolinones, in the presence of **8a**, under different conditions (Table 3).

**Table 3:** Scope of phase transfer catalyzed asymmetric Michael reaction. 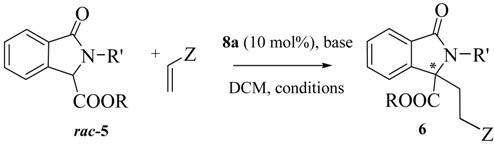

Entry	5	R	R'	Z	T (°C)	t (h)	6	Yield (%) ^a^	ee (%) ^b^
1	**5a**	Et	Bn	COMe	−40	24	**6a**	97	73
2	**5b**	Me	Bn	COMe	−40	24	**6b**	98	70
3	**5c**	*t*-Bu	Bn	COMe	−40	48	**6c**	60	45
4	**5a**	Et	Bn	COEt	−40	24	**6d**	95	58
5	**5a**	Et	Bn	CHO	−40	18	**6e**	90	33
6	**5a**	Et	Bn	CO_2_Me	r.t.	24	-	No reac.	-
7	**5d**	Et	*n*-Bu	CO_2_Me	r.t.	24	**6f**	75	50
8	**5d**	Et	*n*-Bu	CO_2_Me	−20	48	**6f**	75	21
9	**5d**	Et	*n*-Bu	COMe	r.t.	1	**6g**	90	61
10 ^c^	**5d**	Et	*n*-Bu	COMe	r.t.	24	**6g**	95	59
11	**5d**	Et	*n*-Bu	COMe	−20	8	**6g**	95	76
12	**5d**	Et	*n*-Bu	COMe	−40	24	**6g**	96	71
13 ^d^	**5d**	Et	*n*-Bu	COMe	r.t.	4	**6g**	96	38
14	**5d**	Et	*n*-Bu	CN	−20	18	**6h**	97	13
15	**5d**	Et	*n*-Bu	CN	r.t.	5	**6h**	94	36
16	**5e**	Et	H	COMe	−40	8	**6i**	97	20

^a^ Isolated yield. ^b^ Determined by HPLC on chiral column. ^c^ Reaction performed in the presence of Na_2_CO_3_. ^d^ Reaction performed with 10 mol % of PTC **13** instead of **8a**.

**Scheme 1:**
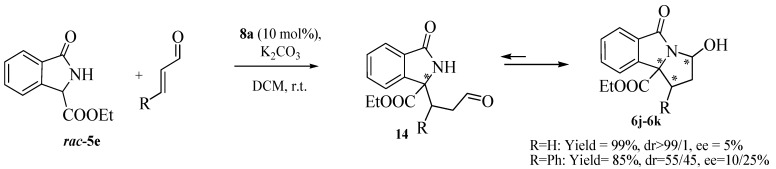
Synthesis of pyrroloisoindolinone analogue.

Isoindolinone **5c** with the hindered *t*-butyl ester group was less effective in terms of reactivity and enantioselectivity than the analogues with ethyl and methyl groups **5a** and **5b**, respectively (Table 3, Entries 1–3). Other Michael acceptors were tested. Ethyl vinyl ketone and acrolein gave the expected adducts in very good yields, but with progressively lower ees than methyl vinyl ketone (Entries 4 and 5). Methyl acrylate did not react with **5a** (Entry 6) and a structural change of the isoindolinone scaffold was necessary to guarantee a higher reactivity.In this case, in the presence of the *n*-butyl substituent on the amide in **5d** instead of a benzyl group, the final adduct was obtained in good yield and moderate ee in a reasonable reaction time (Entries 7 and 8). The isoindolinone **5d**, in the presence of methyl vinyl ketone, slightly affected the enantioselectivity in a positive manner, giving the good value of 76% at −20 °C in very high yield (Entries 9–12). Under these new conditions, Na_2_CO_3_ was slightly less effective than K_2_CO_3_ (entry 10), while the dimeric cinchonidinium salt **13**, synthesized according to reported procedures [40] was less satisfactory than **8a** (entry 13). Acrylonitrile also showed a very good reactivity, with higher enantioselectivity being observed at r.t. (entries 14 and 15). We also tested the isoindolinone **5e** synthesized according to reported procedures [41] in order to investigate the effect of a further structural change on the reactivity and enantioselectivity. Despite the high reactivity toward methyl vinyl ketone, the free NH group was not beneficial for the enantioselectivity of the process, (entry 16). On the other hand the presence of the NH in **5e** was particularly useful because a further cyclization reaction with the acrolein led to the tricyclic derivative **6j** in high yield (Scheme 1). As observed by ^1^H-NMR analysis only one diastereomer was detected, but with very low enantioselectivity. A similar reactivity was observed with cynnamaldehyde, leading to the tricyclic derivative in high yield, but with low enantioselectivity, confirming the negative trend of **5e** with this type of catalysis [42].

Nevertheless, this outcome is particular promising. The obtained compound is an analog of the pyrroloisoindolinone scaffold found, for example, in the cyclin dependent kinase 1,2,4,6 inhibitor [43]. Other studies are in course to improve the enantioselectivity and to enlarge the scope of this class of heterocyclic compounds, also considering eventual transformations of the existing functional groups [42].

In the last part of the discussion we focused on reactions of the Michael acceptor 3-penten-2-one in order to study the diastereoselectivity and possibly to obtain adducts with contiguous tertiary and quaternary stereocenters (Table 4). Also in this case the behavior was rather unexpected. According to the data reported in Table 3, the reactivity was strongly dependent on the isoindolinone structure. Probably due to the congested steric situation at the nucleophilic carbon, *N*-substituted isoindolinones **5a** and **5d** did not react at all, while the substrate **5e** gave smoothly the expected product **6l** with the contiguous quaternary and tertiary stereocenters in high yield and with good diastereoselectivity. Unfortunately, also in this case, a very low enantioselectivity was observed, leaving this challenge to future investigations.

**Table 4:** Reactivity of 3-penten-2-one. 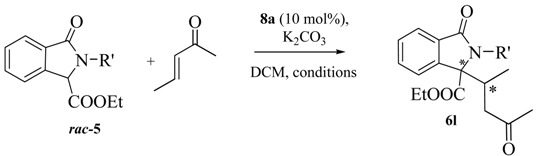

Entry	5	Rʹ	t (h)	T (°C)	Yield (%) ^a^	d.r. ^b^	ee (%) ^c^
1	**5a**	Bn	24	r.t.	-	-	-
2	**5d**	*n*-Bu	24	r.t.	-	-	-
3	**5e**	H	4	r.t.	95	9/1	4
4	**5e**	H	24	−40	92	92/8	8

^a^ Isolated yield. ^b^ Determined by ^1^H-NMR on the crude. ^c^ Determined by HPLC on chiral column.

## 3. Experimental Section 

### 3.1. General Information

All reactions were performed using commercially available compounds without further purification. Column chromatographic purification of products was carried out using silica gel 60 (70–230 mesh, Merck, Darmastdt, Germany). The NMR spectra were recorded on Bruker (Rheinstetten, Germany) DRX 400, 300, 250 spectrometers (400 MHz, 300 MHz, 250 MHz, ^1^H; 100 MHz, 75 MHz, 62.5 MHz ^13^C). Spectra were referenced to residual CHCl_3_ (7.26 ppm, ^1^H, 77.23 ppm, ^13^C). Coupling constants *J* are reported in Hz. Yields are given for isolated products showing one spot on a TLC plate and no impurities detectable in the NMR spectrum. Mass spectral analyses were carried out using a Waters 4 micro quadrupole electrospray spectrometer (Waters, Milford, MA, USA). Elemental analyses were performed with a FLASHEA 1112 series for CHNS-O apparatus (Thermo Scientific, Waltham, MA, USA). Polarimeter Jasco P-2000 (Tokio, Japan), HPLC Waters dual 1485 (Waters).

### 3.2. Synthesis 

The isoindolinones **5a**–**e** were synthesized according to reported procedures and spectroscopic were in agreement with literature [29,41,44]. Only **5c** and **5d** have never been described and the corresponding spectroscopic data are given below.

*tert-Butyl-2-benzyl-3-oxoisoindoline-1-carboxylate* (**5c**). To a solution of compound **5b** (56 mg, 0.2 mmol) in absolute EtOH (1 mL), NaOH 0.5 M (1 mL) was added and the mixture was stirred for 2 h at room temperature. The solvent was removed and the residue was taken up with water, acidified to pH 1 and extracted twice with dichloromethane. The organic layers were combined and evaporated to give a white solid (52 mg, 0.18 mmol) which was resolubilized in dichloromethane (1 mL). Then EDC (40 mg, 0.204 mmol), DMAP (2 mg, 0.02 mmol) and *tert*-butanol (30 μL, 0.670 mmol, in excess) were added to the solution and the mixture was allowed to stir at room temperature for 3 h. Purification by chromatography (ethyl acetate–hexane 1:4) gave a waxy solid. Yield: 52%. ^1^H-NMR (400 MHz, CDCl_3_) δ: 7.89 (d, 1H, *J* = 6.5 Hz), 7.35–7.52 (m, 4H), 7.34–7.27 (m, 4H), 5.49 (d, 1H, *J* = 16 Hz), 4.76 (s, 1H), 4.27 (d, 1H, *J* = 16 Hz), 1.47 (s, 9H). MS (ESI): *m/z* = 324 (M+H)^+^. Anal. Calcd. for C_20_H_21_NO_3_ C, 74.28; H, 6.55; N, 4.33; found C, 74.33; H, 6.46; N, 4.30.

*Ethyl 2-butyl-3-oxoisoindoline-1-carboxylate* (**5d**). Yield 85%; oil. ^1^H-NMR (400 MHz, CDCl_3_) δ: 7.99–7.95 (m, 1H), 7.74–7.45 (m, 3H), 5.31 (s, 1H), 4.47–4.24 (m, 2H), 4.23–4.15 (m, 1H), 3.43–3.32 (m, 1H), 1.79–1.69 (m, 2H), 1.54–1.35 (m, 5H), 1.07 (t, 3H, *J* = 7.2 Hz). ^13^C-NMR (100 MHz, CDCl_3_) δ: 168.7, 168.5, 139.5, 132.2, 131.9, 129.3, 124.0, 122.7, 62.3, 62.2, 41.3, 30.2, 20.3, 14.3, 13.9. MS (ESI): *m/z* = 262 (M+H)^+^. Anal. calcd for C_15_H_19_NO_3_ C, 68.94; H, 7.33; N, 5.36; found C, 68.83; H, 7.46; N, 5.30.

### 3.3. Procedure for Preparation of rac-**6**

The Michael acceptor (1.5 eq) was added at r.t. to a solution of isoindolinones **5** (0.05 mmol) and K_2_CO_3_ (0.5 eq) in CH_3_CN (1 mL). The reaction was stirred overnight, the solvent was evaporated and the mixture purified directly on a chromatographic column eluting with 4:1 hexane–ethyl acetate, to afford *rac*-**6** in yields ranging from 70%–90% [45]. 

### 3.4. Procedure for Enantioselective Michael Reactions of **5** to Afford **6**

The Michael acceptor (1.5 eq) was added at −40 °C to a stirred solution of **5** (0.1 mmol, 1 eq.), K_2_CO_3_ (0.1 mmol, 1 eq.) and **8a** (10% mol) in CH_2_Cl_2_ (1.5 mL). The reaction was monitored by TLC until the disappearance of **5**. Then, the mixture was purified directly by flash chromatography eluting with 4:1 hexane–ethyl acetate to affording compounds **6** as waxy solids in yields ranging from 60%–99%. 

*Ethyl 2-benzyl-3-oxo-1-(3-oxobutyl)isoindoline-1-carboxylate* (**6a**). Yield: 35 mg (97%); ${\left[\text{α}\right]}_{D}^{20}$ *=* −2.0 (c = 0.1 M CHCl_3_); ee: 73%; ^1^H-NMR (400 MHz, CDCl_3_) δ: 7.91 (d, 1H, *J* = 8 Hz,), 7.55–7.46 (m, 2H), 7.4–7.36 (m, 3H), 7.27 (q, 3H, *J* = 7.6 Hz), 5.1 (d, 1H, *J* = 15.2 Hz,), 4.3 (d, 1H, *J* = 15.2 Hz,), 4.0 (q, 2H, *J* = 7.2 Hz), 2.74–2.67 (m, 1H), 2.39–2.35 (m, 1H), 1.61 (s, 3H), 1.48–1.41 (m, 2H), 1.09 (t, 3H, *J* = 7.0 Hz); ^13^C-NMR (100 MHz, CDCl_3_) δ: 206.5, 169.8, 169.1, 142.9, 137.2, 132.2, 131.5, 129.2, 129.0, 128.4, 127.6, 123.9, 121.4, 71.6, 62.1, 44.7, 35.9, 29.4, 26.2, 13.7; MS (ESI): *m/z* = 366 (M+H)^+^. Anal. calcd for C_22_H_23_NO_4_ C, 72.31; H, 6.34; N, 3.83; found C, 72.20; H, 6.45; N, 3.70. Chiral HPLC: ODH column, hexane–*i*PrOH (4:1), flow: 0.6 mL/min, t: 15.6 min and 17.5 min.

*Methyl 2-benzyl-3-oxo-1-(3-oxobutyl)isoindoline-1-carboxylate* (**6b**). Yield: 34 mg (98%); ${\left[\text{α}\right]}_{D}^{20}$ *=* −2.7 (c = 0.7 M CHCl_3_); ee: 70%; ^1^H-NMR (400 MHz, CDCl_3_) δ: 7.91 (d, 1H, *J* = 7.4 Hz), 7.54–7.36 (m, 3H), 7.28–7.22 (m, 5H), 4.98 (d, 1H, *J* = 14.9 Hz), 4.42 (d, 1H, *J* = 15.1 Hz), 3.46 (s, 3H), 2.77–2.65 (m, 1H), 2.48–2.42 (m, 1H), 1.65 (s, 3H), 1.53–1.43 (m, 2H); ^13^C-NMR (100 MHz, CDCl_3_) δ: 206.7, 170.7, 169.3, 143.2, 137.4, 132.6, 131.9, 129.5, 129.4, 128.8, 127.9, 124.3, 121.8, 71.6, 53.1, 44.9, 36.2, 29.7, 26.4; MS (ESI): *m/z* = 352 (M+H)^+^. Anal. calcd for C_21_H_21_NO_4_ C, 71.78; H, 6.02; N, 3.99; found C, 71.60; H, 6.11; N, 3.87; Chiral HPLC: ODH column, hexane–*i*PrOH (4:1), flow: 0.6 mL/min, t: 19.2 min and 22.6 min.

*tert-Butyl 2-benzyl-3-oxo-1-(3-oxobutyl)isoindoline-1-carboxylate* (**6c**). Yield: 24 mg (60%); ${\left[\text{α}\right]}_{D}^{20}$ *=* −56.0 (c = 0.8 M CHCl_3_); ee: 45%; ^1^H-NMR (400 MHz, CDCl_3_) δ: 7.87 (d, 1H, *J* = 6.7 Hz), 7.85–7.35 (m, 3H), 7.28–7.18 (m, 5H), 5.18 (d, 1H, *J* = 15.5 Hz), 4.23 (d, 1H, *J* = 15.0 Hz), 2.66–2.30 (m, 1H), 2.29–2.14 (m, 1H), 1.5 (m, 5H), 1.28 (s, 9H); ^13^C-NMR (100 MHz, CDCl_3_) δ: 206.7, 169.3, 168.7, 143.3, 137.5, 132.1, 131.6, 129.1, 128.5, 127.5, 123.9, 121.4, 83.1, 72.5, 44.9, 36.2, 29.6, 27.5, 26.3; MS (ESI): *m/z* = 394 (M+H)^+^. Anal. calcd for C_24_H_27_NO_4_ C, 73.26; H, 6.92; N, 3.56; found C, 73.40; H, 6.80; N, 3.65; Chiral HPLC: ODH column, hexane–*i*PrOH (4:1), flow: 0.6 mL/min. t: 13.9 min and 14.8 min.

*Ethyl 2-benzyl-3-oxo-1-(3-oxopentyl)isoindoline-1-carboxylate* (**6d**). Yield: 36 mg (95%); ${\left[\text{α}\right]}_{D}^{20}$ *=* −1.8 (0.1 M, CHCl_3_); ee: 58%; ^1^H-NMR (400 MHz, CDCl_3_) δ: 7.89 (d, 1H, *J* = 1.5 Hz), 7.87–7.43 (m, 3H), 7.38–7.35 (m, 1H), 7.27–7.20 (m, 4H), 5.06 (d, 1H, *J* = 15.3 Hz), 4.31 (d, 1H, *J* = 15.3 Hz), 3.97–3.92 (m, 2H), 2.72–2.67 (m, 1H), 2.44–2.33 (m, 1H), 1.80–1.76 (m, 2H), 1.45–1.36 (m, 2H), 1.08–1.02 (m, 3H), 0.72 (t, 3H, *J* = 7.3 Hz); ^13^C-NMR (100 MHz, CDCl_3_) δ: 209.6, 170.2, 169.5, 143.7, 137.9, 132.5, 131.9, 129.5, 129.4, 128.7, 127.8, 124.2, 121.9, 72.0, 62.4, 45.1, 35.7, 35.0, 26.5, 14.0, 8.2; MS (ESI): *m/z* = 380 (M+H)^+^. Anal. calcd for C_23_H_25_NO_4_ C, 72.80; H, 6.64; N, 3.69; found C, 72.95; H, 6.60; N, 3.59; Chiral HPLC: ODH column, hexane–*i*PrOH (4:1), flow: 0.6 mL/min, t: 13.1 min and 14.5 min.

*Ethyl 2-benzyl-3-oxo-1-(3-oxopropyl)isoindoline-1-carboxylate* (**6e**).Yield: 32 mg (90%); ${\left[\text{α}\right]}_{D}^{20}$ *=* +0.52 (0.9 M, CHCl_3_); ee: 33%; ^1^H-NMR (300 MHz, CDCl_3_) δ: 9.10 (s, 1H), 7.89–7.86 (m, 1H), 7.88–7.41 (m, 6H), 7.37–7.20 (m, 2H), 5.07 (d, 1H, *J* = 15.2 Hz), 4.31 (d, 1H, *J* = 15.2 Hz), 4.01–3.88 (m, 2H), 2.75–2.65 (m, 1H), 2.44–2.34 (m, 1H), 1.48 (t, 2H, *J* = 7.7 Hz), 1.05 (t, 3H, *J* = 7.1 Hz); ^13^C-NMR (75 MHz, CDCl_3_) δ: 200.0, 170.1, 169.4, 143.0, 137.4, 132.6, 131.9, 129.7, 129.3, 128.8, 128.0, 124.4, 121.6, 71.7, 62.5, 45.1, 37.2, 24.8, 14.0; MS (ESI): *m/z* = 352 (M+H)^+^. Anal. calcd for C_21_H_21_NO_4_ C, 71.78; H, 6.02; N, 3.99, found C, 71.90; H, 6.12; N, 3.87; Chiral HPLC: ODH column, hexane–*i*PrOH (4:1), f: 0.6 mL/min., t: 21.4 min and 23.6 min. 

*Ethyl 2-butyl-1-(3-methoxy-3-oxopropyl)-3-oxoisoindoline-1-carboxylate* (**6f**).Yield: 26 mg (75%); ${\left[\text{α}\right]}_{D}^{20}$ = −25.3 (0.7 M, CHCl_3_); ee: 50%; ^1^H-NMR (250 MHz, CDCl_3_) δ: 7.82 (d, 1H, *J* = 6.5 Hz), 7.54–7.40 (m, 3H), 4.19–4.08 (m, 2H), 3.55 (s, 3H), 3.47–3.32 (m, 2H), 2.94–2.84 (m, 1H), 2.62–2.51 (m, 1H), 1.95–1.83 (m, 1H), 1.73–1.62 (m, 3H), 1,4 (q, 2H, *J* = 7.3 Hz), 1.15 (t, 3H, *J* = 7.1 Hz). 0.92 (t, 3H, *J* = 7.1 Hz); ^13^C-NMR (60 MHz, CDCl_3_) δ: 172.6, 170.2, 169.1, 142.5, 132.1, 131.9, 129.2, 123.7, 121.4, 71.3, 62.2, 51.6, 41.5, 30.3, 27.5, 27.4, 20.5, 13.7, 13.6; MS (ESI): *m/z* = 348 (M+H)^+^. Anal. calcd for C_19_H_25_NO_5_ C, 65.69; H, 7.25; N, 4.03; found C, 65.80; H, 7.40; N, 4.09; Chiral HPLC: ODH column, hexane–*i*PrOH (4:1), flow: 0.6 mL/min, t: 29.2 min and 33.7 min.

*Ethyl 2-butyl-3-oxo-1-(3-oxobutyl)isoindoline-1-carboxylate* (**6g**). Yield: 31 mg (95%); ${\left[\text{α}\right]}_{D}^{20}$ *=* −1.4 (0.1 M CHCl_3_); ee: 76%; ^1^H-NMR (400 MHz, CDCl_3_) δ: 7.84 (d, 1H, *J* = 7.4 Hz), 7.56–7.47 (m, 2H), 7.40 (d, 1H, *J* = 7.1 Hz), 4.18–4.09 (m, 2H), 3.44–3.36 (m, 1H), 3.34–3.31 (m, 1H), 2.86–2.78 (m, 1H), 2.54–2.47 (m, 1H), 2.03–1.94 (m, 1H), 1.75 (s, 3H), 1.73–1.68 (m, 2H), 1.61–1.55 (m, 1H), 1.39 (q, 2H, *J* = 7.5 Hz), 1.16 (t, 3H, *J* = 7.1 Hz), 0.94 (t, 3H, *J* = 7.3 Hz); ^13^C-NMR (100 MHz, CDCl_3_) δ: 206.6, 170.2, 169.1, 142.8, 132.1, 132.0, 129.2, 123.6, 121.6, 71.3, 62.1, 41.5, 36.4, 30.3, 29.9, 25.9, 20.5, 13.8, 13.6; MS (ESI): *m/z* = 332 (M+H)^+^. Anal. calcd for C_19_H_25_NO_4_ C, 68.86; H, 7.60; N, 4.23; C, 68.94; H, 7.75; N, 4.15; Chiral HPLC: IA-3 column, hexane–*i*PrOH (4:1), f: 0.6 mL/min, t: 11.3 min and 15.9 min.

*Ethyl 2-butyl-1-(2-cyanoethyl)-3-oxoisoindoline-1-carboxylate* (**6h**).Yield: 29 mg (97%); ${\left[\text{α}\right]}_{D}^{20}$ *=* −10.1 (1.6 M CHCl_3_); ee: 36%; ^1^H-NMR (250 MHz, CDCl_3_) δ: 7.86–7.83 (m, 1H), 7.84–7.50 (m, 2H), 7.44–7.41 (m, 1H), 4.20–4.05 (m, 2H), 3.51–3.42 (m, 1H), 3.37–3.27 (m, 1H), 2.98–2.86 (m, 1H), 2.65–2.53 (m, 1H), 1.99–1.86 (m, 1H), 1.73–1.65 (m, 2H), 1.41 (q, 2H, *J* =7.2 Hz), 1.25 (m, 1H), 1.15 (t, 3H, *J* = 7.1 Hz), 0.92 (t, 3H, *J* = 7.1 Hz); ^13^C-NMR (60 MHz, CDCl_3_) δ: 169.6, 168.9, 141.4, 132.5, 132.2, 129.9, 124.2, 121.2, 118.1, 70.7, 62.6, 60.3, 41.6, 30.5, 28.5, 20.5, 13.7, 11.0; MS (ESI): *m/z* = 315 (M+H)^+^. Anal. calcd for C_18_H_22_N_2_O_3_ C, 68.77; H, 7.05; N, 8.91; C, 68.90; H, 7.14; N, 8.82; Chiral HPLC: IE-3 column, hexane–*i*PrOH (4:1), f: 0.6 mL/min. t: 10.0 min and 11.4 min.

*Ethyl 3-oxo-1-(3-oxobutyl)isoindoline-1-carboxylate* (**6i**).Yield: 25 mg (97%); ${\left[\text{α}\right]}_{D}^{20}$ *=* −0.3 (0.5 M CHCl_3_); ee: 20%; ^1^H-NMR (400 MHz, CDCl_3_) δ: 7.82 (d, 1H, *J* = 7.28 Hz), 7.63–7.51 (m, 3H), 6.68 (brs, 1H), 4.22 (q, 2H, *J* = 6.6 Hz), 2.56–2.25 (m, 2H), 2.19–2.12 (m, 2H), 2.03 (s, 3H), 1.26 (t, 3H, *J* = 7.1 Hz);. ^13^C-NMR (100 MHz, CDCl_3_) δ: 206.8, 170.3, 169.8, 144.1, 132.6, 131.0, 129.3, 123.8, 123.3, 67.0, 62.4, 37.1, 30.9, 29.9, 13.9; MS (ESI): *m/z* = 276 (M+H)^+^. Anal. calcd for C_15_H_17_NO_4_ C, 65.44; H, 6.22; N, 5.09; C, 65.54; H, 6.34; N, 5.15; Chiral HPLC: IA3 column, hexane–*i*PrOH (4:1), flow 0.6 mL/min. t: 19.2 min and 29.4 min.

*Ethyl 2,3,5,9b-tetrahydro-3-hydroxy-5-oxo-1H-pyrrolo[2,1-a]isoindole-9b-carboxylate* (**6j**). Yield: 26 mg (99%); ${\left[\text{α}\right]}_{D}^{20}$ *=* −0.1 (1 M CHCl_3_); ee: 5%; ^1^H-NMR (300 MHz, CDCl_3_) δ: 7.77 (d, 1H, *J* = 7.1 Hz), 7.61–7.45 (m, 3H), 5.71 (q, 1H, *J* = 6.3 Hz), 4.26–4.21 (m, 2H), 3.63 (d, 1H, *J* = 6.1 Hz), 2.80–2.75 (m, 1H), 2.68–2.63 (m, 1H), 2.24–2.18 (m, 1H), 1.72–1.67 (m, 1H), 1.25 (t, 3H, *J* = 6.9 Hz); ^13^C-NMR (100 MHz, CDCl_3_) δ: 171.4, 171.0, 145.1, 133.2, 131.9, 129.7, 124.9, 123.3, 80.2, 75.5, 62.7, 37.9, 34.2, 14.2; MS (ESI): *m/z* = 262 (M+H)^+^. Anal. calcd for C_14_H_15_NO_4_: C, 64.36; H, 5.79; N, 5.36; found: C, 64.51; H, 5.65; N, 5.43; Chiral HPLC: colonna IE-3 column, hexane–*i*PrOH (4:1) flow: 0.6 mL/min. t: 32.2 min and 37.6 min.

*Ethyl 2,3,5,9b-tetrahydro-3-hydroxy-5-oxo-1-phenyl-1H-pyrrolo[2,1-a]isoindole-9b-carboxylate* (**6k**). The title compound was obtained as a mixture of two diastereomers. Yield: 29 mg (85%). ee: 10/25%. ^1^H-NMR (300 MHz, CDCl_3_) δ: 7.85–7.82 (m, 1H, minor diast.), 7.59–7.55 (m, 1H), 7.43–7.40 (m, minor diast), 7.29–7.22 (m, 3H), 7.10–6.98 (m, 3H), 6.79 (dd, 2H, *J_2_* = 1.5 Hz, *J_1_* = 6.2 Hz), 6.12 (q, 1H, *J* = 6.4 Hz), 5.82 (t, 1H, *J* = 5.3 Hz, minor diast.), 4.39–4.20 (m, 3H), 3.99 (m, minor diast.), 3.64 (d, 1H, *J* = 6.3 Hz), 3.13 (q, *J* = 7.0 Hz, minor diast.), 2.90 (ddd, 1H, *J_3_* = 2.6 Hz, *J_2_* = 6.7 Hz, *J_1_* = 12 Hz), 2.70 (ddd, 1H, *J_3_* = 4.7 Hz, *J_2_* = 7.7 Hz, *J_1_* = 13 Hz), 1.35 (t,3H, *J* = 7.1 Hz) 0.93 (t, 3H, *J* = 7.1 Hz, minor diast.); ^13^C-NMR (75 MHz, CDCl_3_) δ: 171.7, 170.0, 143.3, 141.9, 138.2, 134.7, 132.2, 132.0, 129.5, 129.0, 128.6, 128.2, 127.9, 127.7, 127.0, 125.4, 124.3, 124.0, 79.4, 79.0, 62.7, 62.3, 54.5, 50.0, 44.5, 43.2, 14.0, 13.2; MS (ESI): *m/z* = 338 (M+H)^+^. Anal. calcd for C_20_H_19_NO_4_: C, 71.20; H, 5.68; N, 4.15; found: C, 71.32; H, 5.77; N, 4.02; Chiral HPLC: IA-3 column, hexane–*i*PrOH (9:1), flow: 0.6 mL/min. Major diast. t: 12.5 min and 14.2 min. Minor diast. t: 16 min and 19.4 min.

*Ethyl 3-oxo-1-(4-oxopentan-2-yl)isoindoline-1-carboxylate* (**6l**). Yield: 27 mg (95%); ^1^H-NMR (400 MHz, CDCl_3_) δ: 7.81 (d, 1H, *J* = 7.5 Hz), 7.69 (d, 1H, *J* = 7.7 Hz), 7.60 (t, 1H, *J* = 7.5 Hz), 7.53 (t, 1H*, J* = 7.5 Hz), 7.18 (brs, 1H), 7.07 (brs, 1H, minor diastereomer), 4.22–4.16 (m, 2H), 3.19–3.14 (m, 1H), 2.50 (dd, *J* = 16.8 Hz, 3.6.Hz, 1H), 2.41 (dd, *J* = 16.8 Hz, 9.2 Hz, 1H), 2.17 (s, 3H), 1.87 (s, 1H, minor diastereomer), 1.26–1.19 (m, 3H), 1.08 (d, 2H, *J* = 9.2 Hz), 0.59 (d, 3H*, J* = 6.7 Hz); ^13^C-NMR (100 MHz, CDCl_3_) δ: 206.6, 170.9, 170.8, 144.4, 132.7, 131.6, 129.5, 123.8, 123.7, 71.5, 62.6, 46.3, 35.7, 30.9, 14.2, 13.6; MS (ESI): *m/z* = 290 (M+H)^+^. Anal. calcd for C_16_H_19_NO_4_: C, 64.42; H, 6.62; N, 4.84; found: 64.55; H, 6.52; N, 4.71; Chiral HPLC: IE-3 column, hexane–*i*PrOH (4:1), f: 0.8 mL/min. Major diast. t: 8.9.5 min and 13.0 min. Minor diast. t: 9.7 min and 16.4 min.

## 4. Conclusions 

In conclusion, 3-substituted isoindolinones have been used for the first time as nucleophiles in asymmetric Michael reactions under phase transfer catalyzed conditions in the synthesis of adducts with tetrasubstituted stereocenters. Several electron-deficient olefins were tested. Excellent chemical yields were obtained in the presence of chiral phase transfer catalysts, while organocatalysts were less effective. However, variable enantioselectivities were observed, with good values only occurring in the presence of methyl vinyl ketone. Other studies are in course with the aim to enlarge the substrate scope and field of application of these isoindolinone-based nucleophiles in different asymmetric reactions.

## Supplementary Materials

Supplementary materials are available at: http://www.mdpi.com/1420-3049/20/05/8484/s1.
