# Supplementary material for: Isoindolinones as Michael Donors under Phase Transfer Catalysis: Enantioselective Synthesis of Phthalimidines Containing a Tetrasubstituted Carbon Stereocenter
Source: Molecules. 2015 May 12;20(5):8484–98. doi: 10.3390/molecules20058484 (PMC6272520; doi:10.3390/molecules20058484)
Supplement: Supplementary file 1 [file molecules-20-08484-s001.pdf]

Supplementary Materials

1. Copies of NMR Spectra of Most Relevant Key Intermediates and Final Adducts

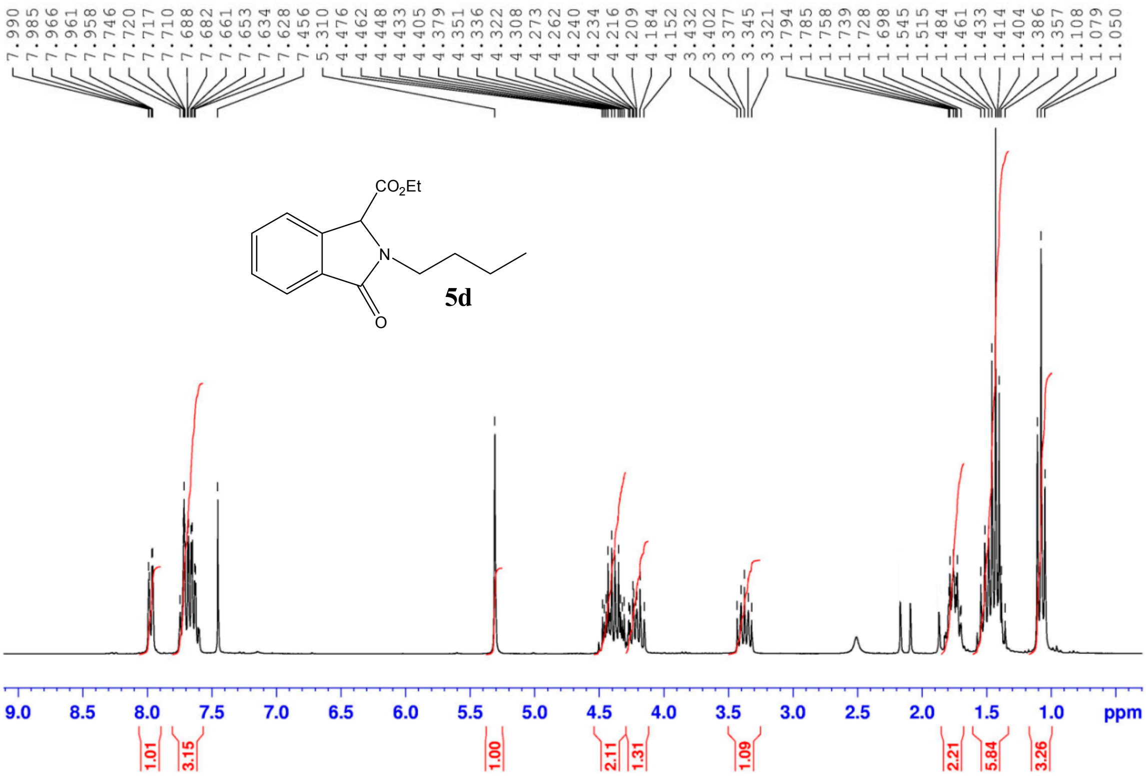

Figure S1. ^{1}H-NMR of compound **5d**.

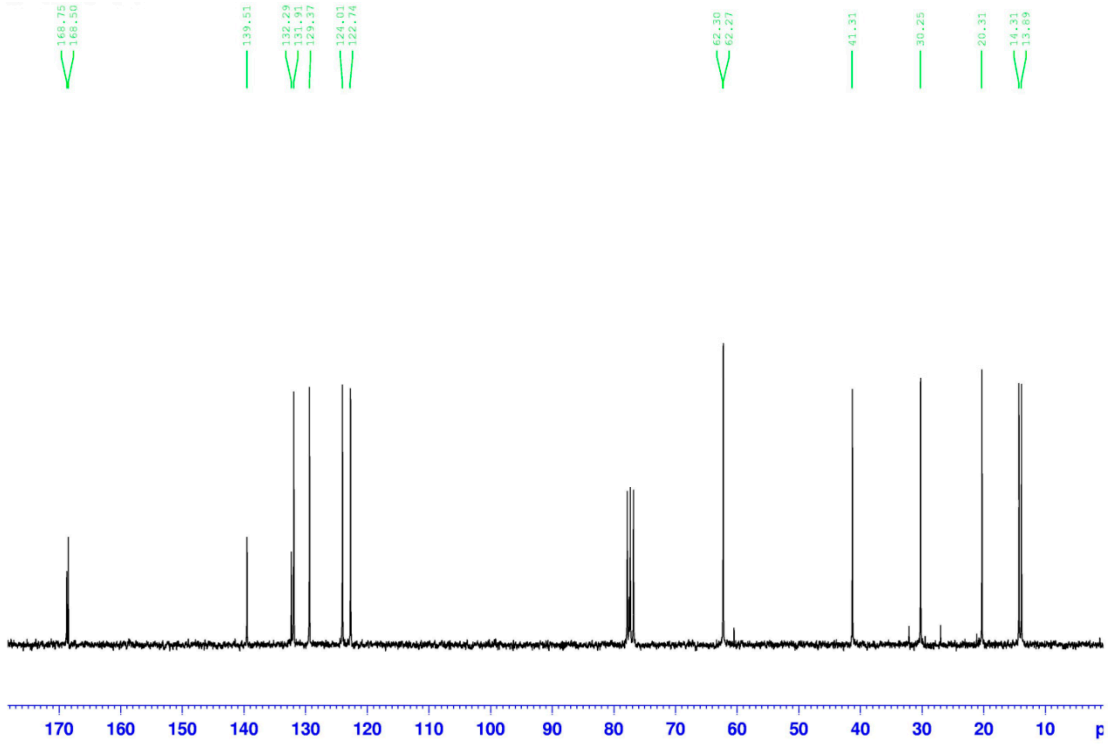

Figure S2. ^{13}C-NMR of compound **5d**.

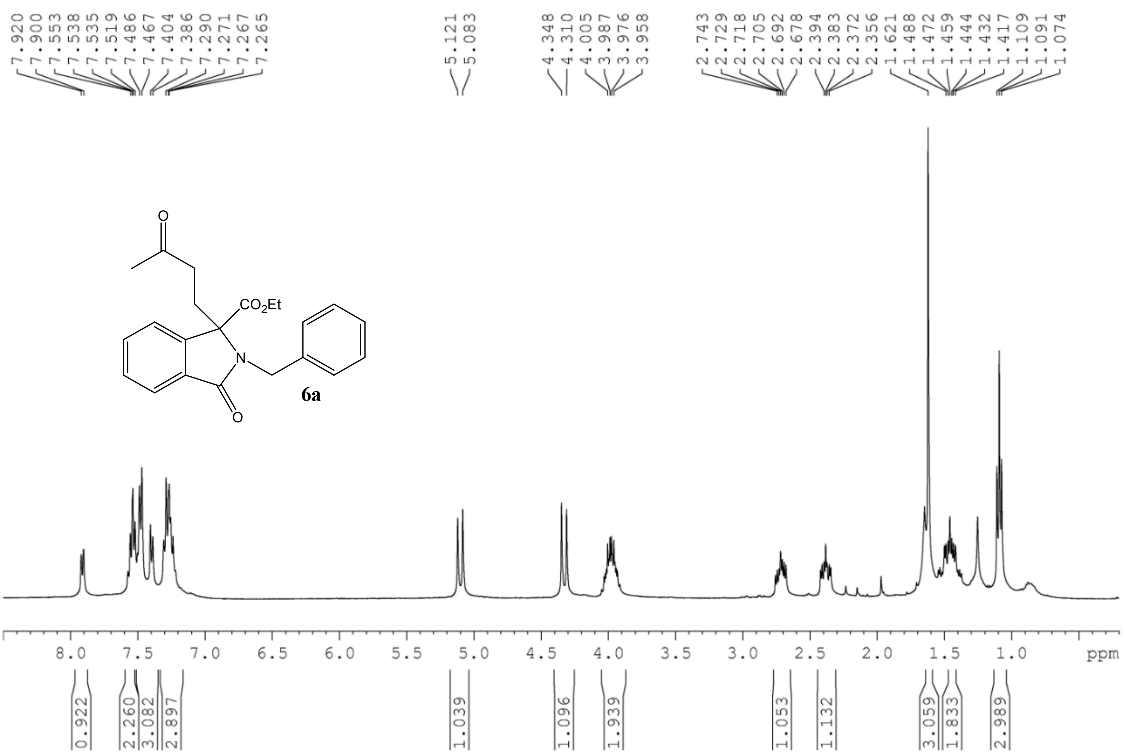

Figure S3. ^{1}H-NMR of compound **6a**.

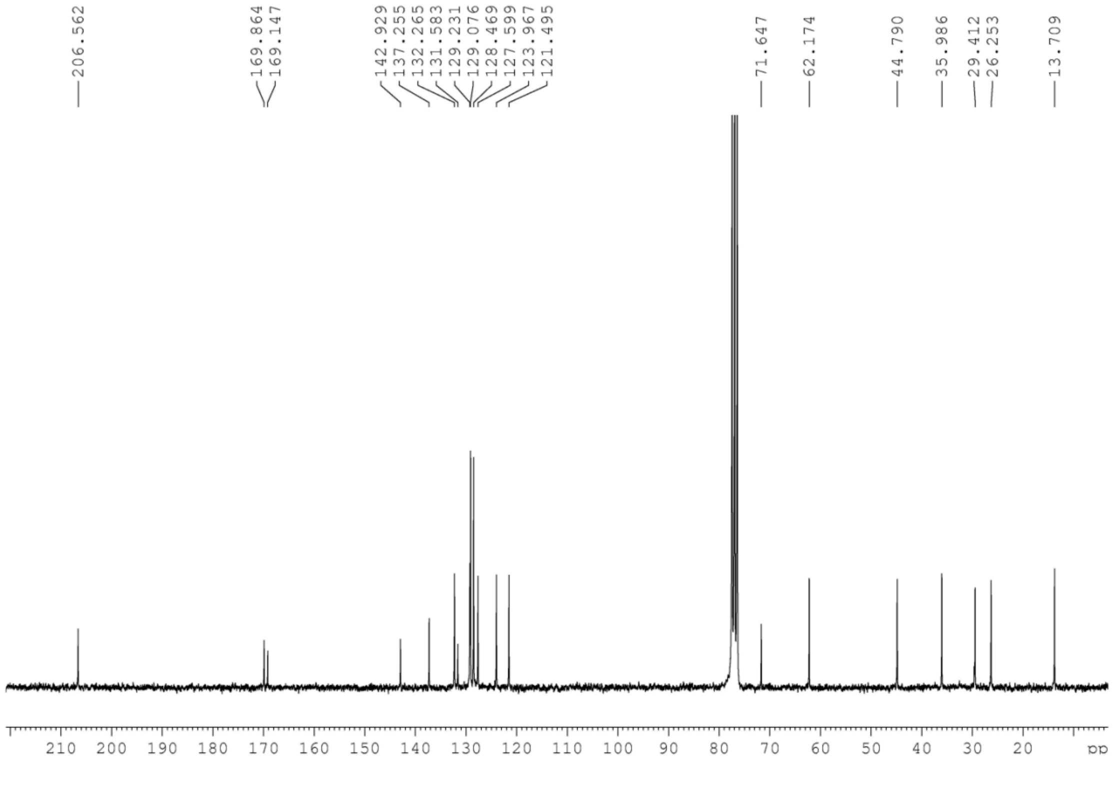

Figure S4. ^{13}C-NMR of compound **6a**.

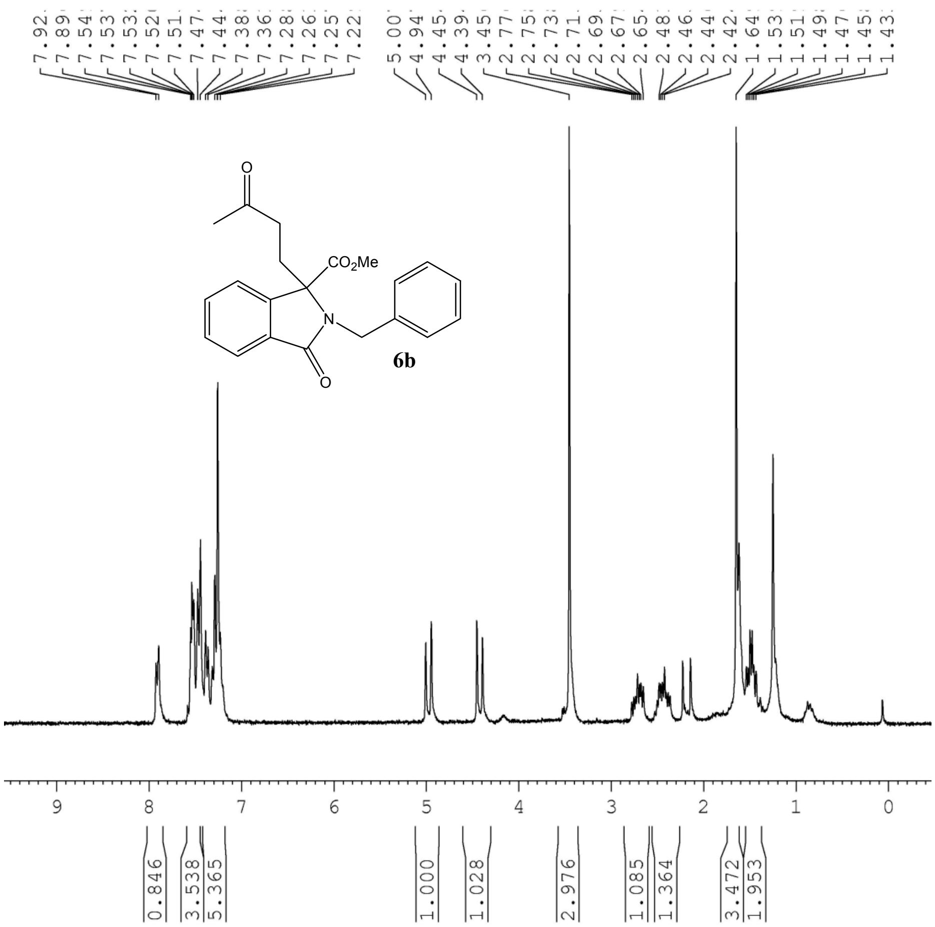

Figure S5. ^{1}H-NMR of compound **6b**.

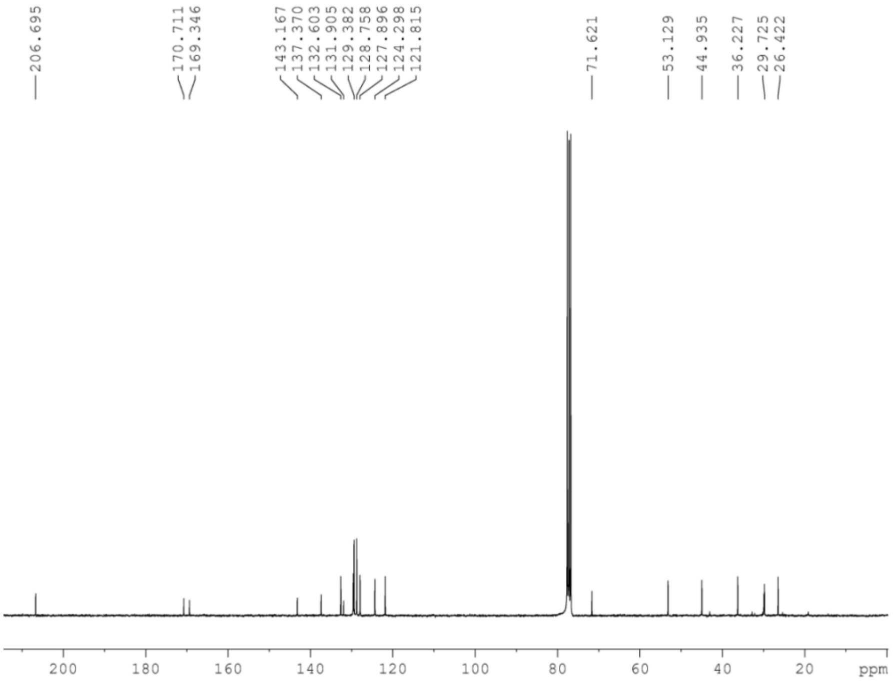

Figure S6. ^{13}C-NMR of compound **6b**.

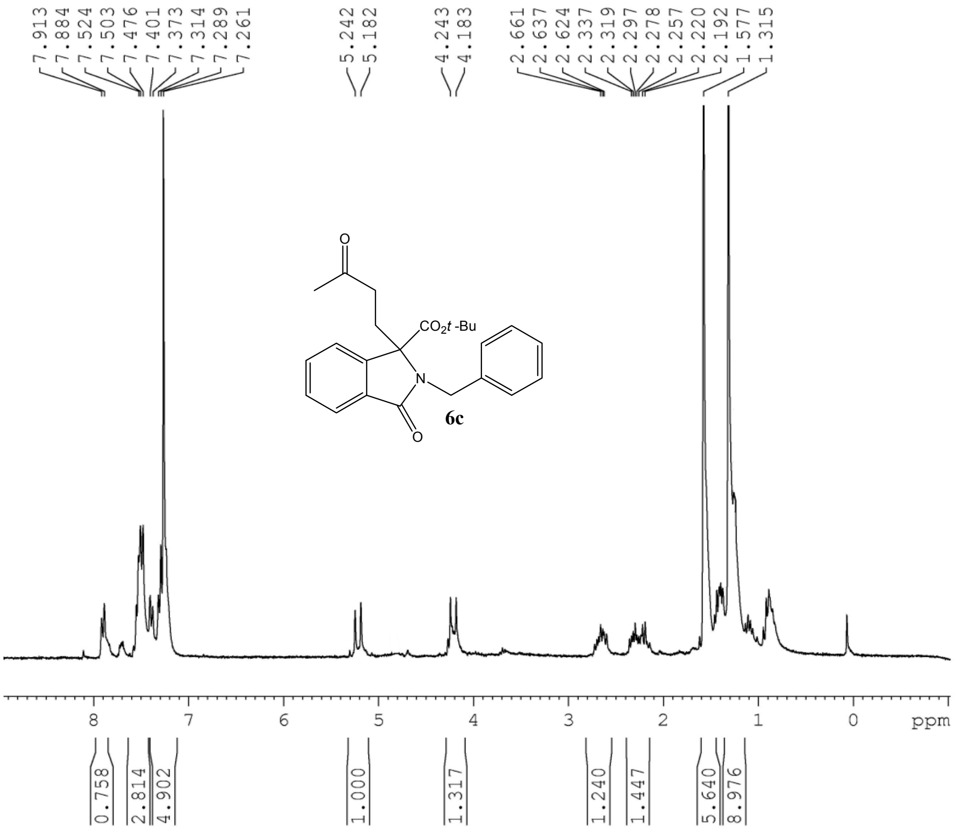

Figure S7. ^{1}H-NMR of compound **6c**.

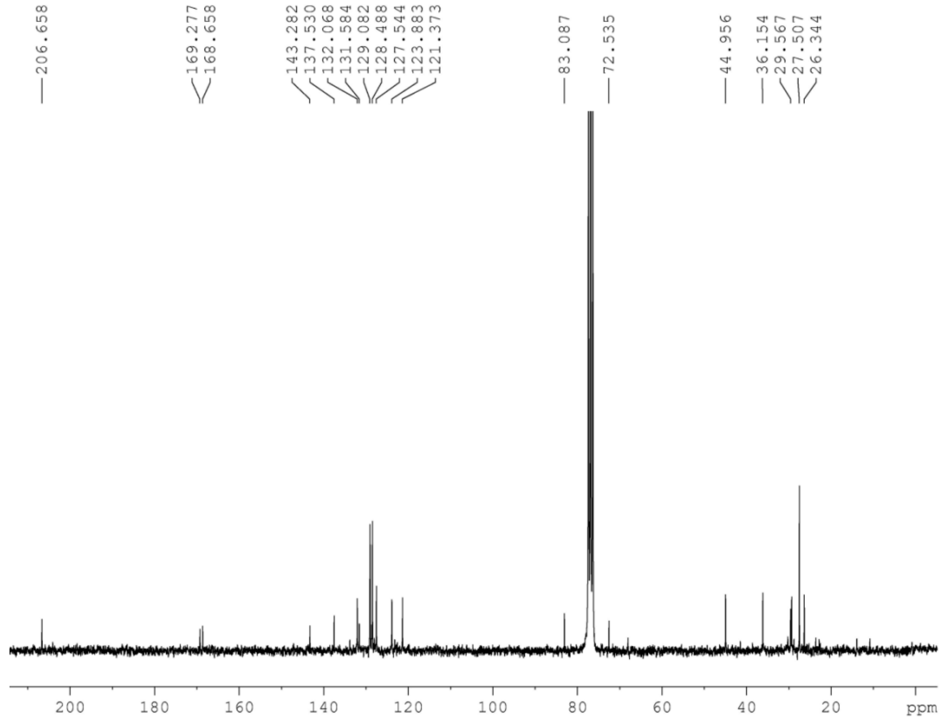

Figure S8. ^{13}C-NMR of compound **6c**.

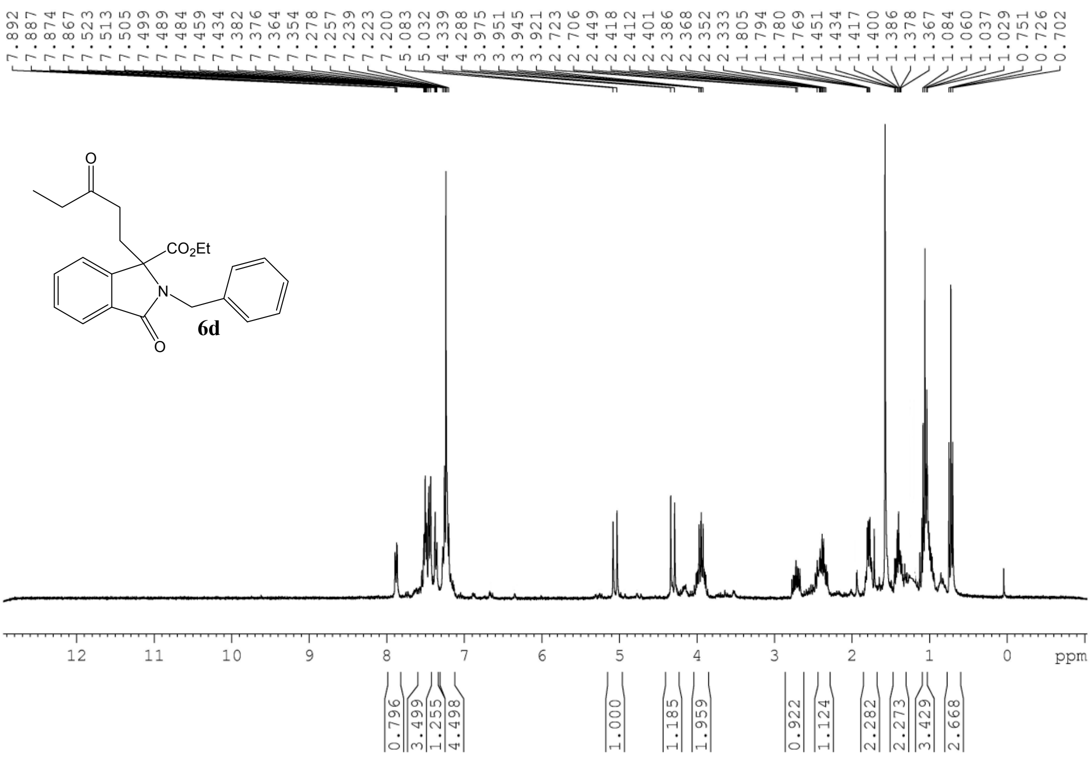

Figure S9. ^{1}H-NMR of compound **6d**.

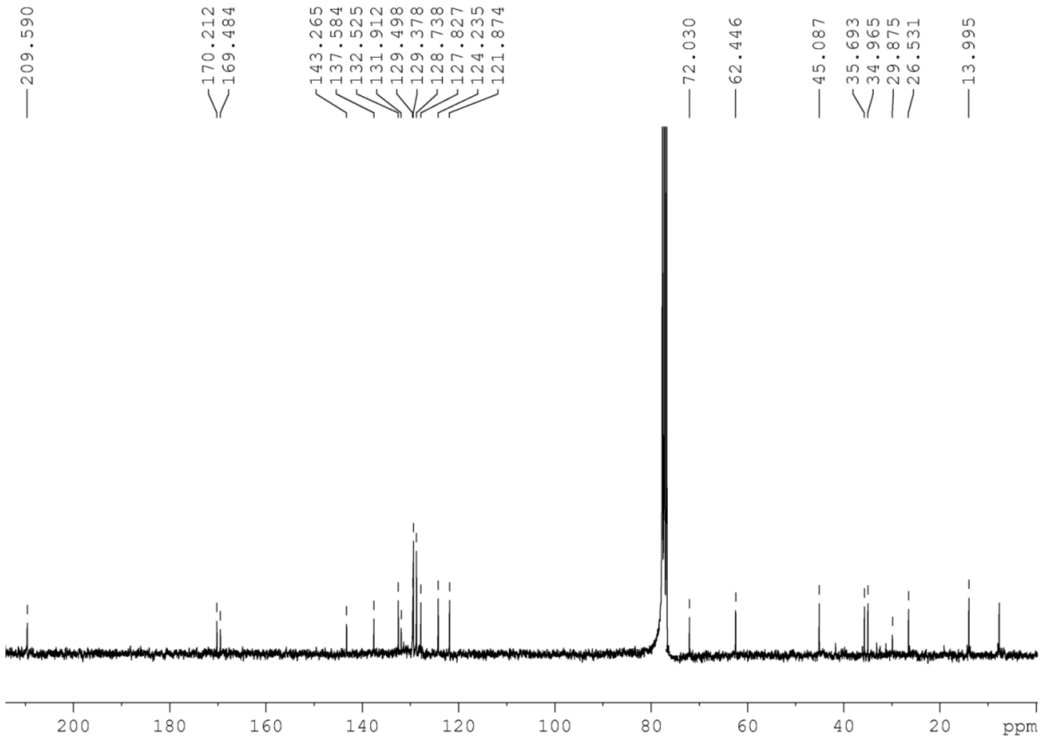

Figure S10. ^{13}C-NMR of compound **6d**.

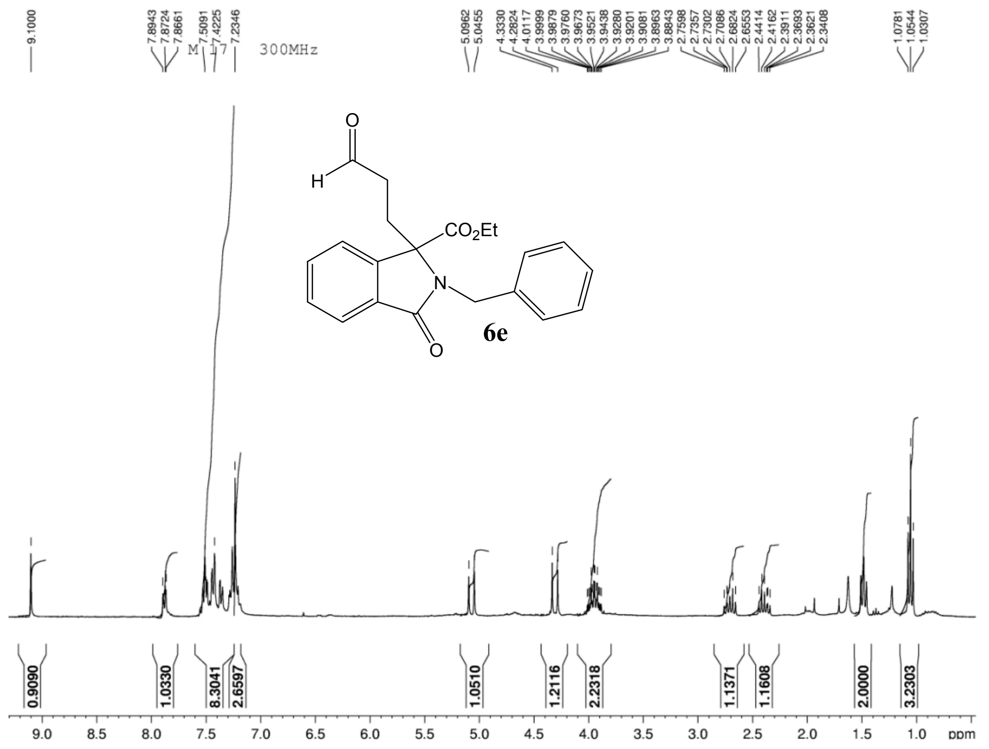

Figure S11. ^{1}H-NMR of compound **6e**.

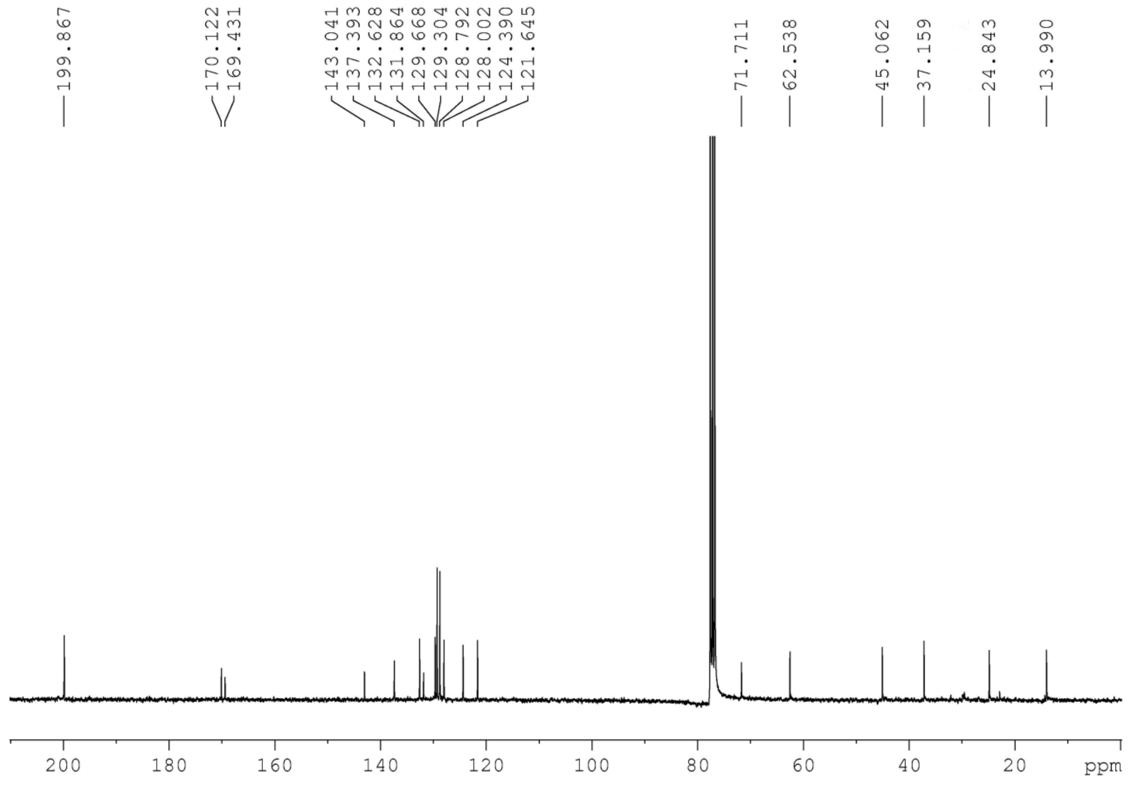

Figure S12. ^{13}C-NMR of compound **6e**.

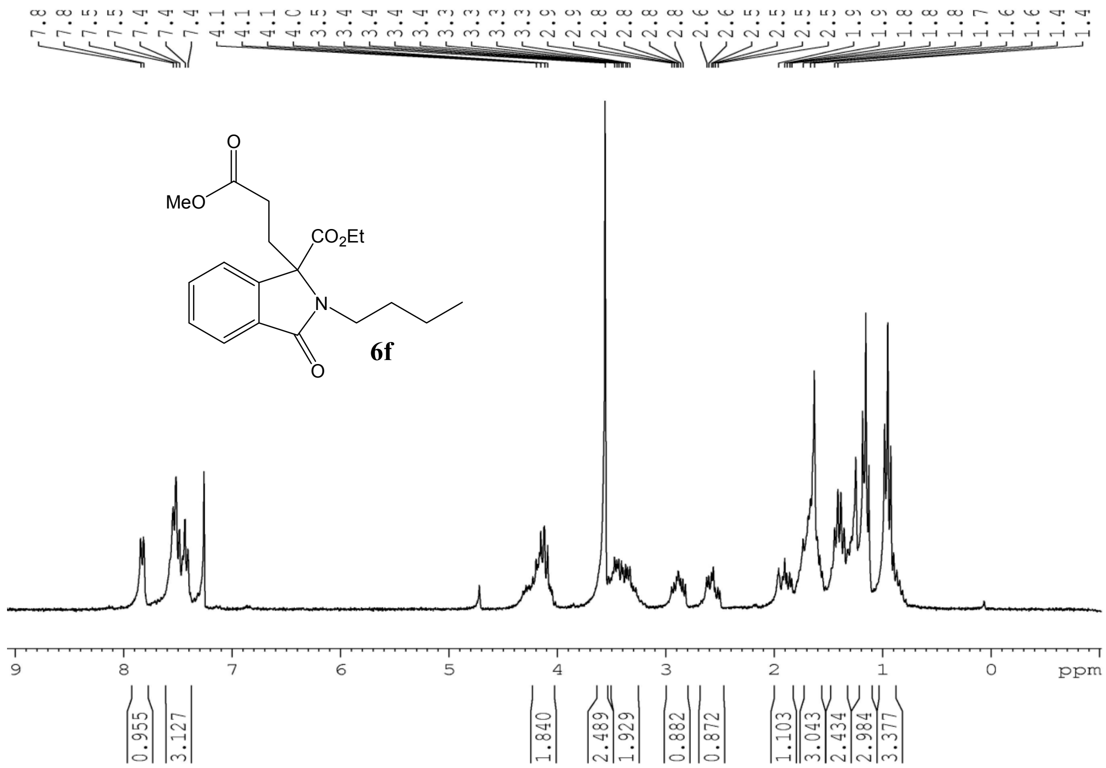

Figure S13. ^{1}H-NMR of compound **6f**.

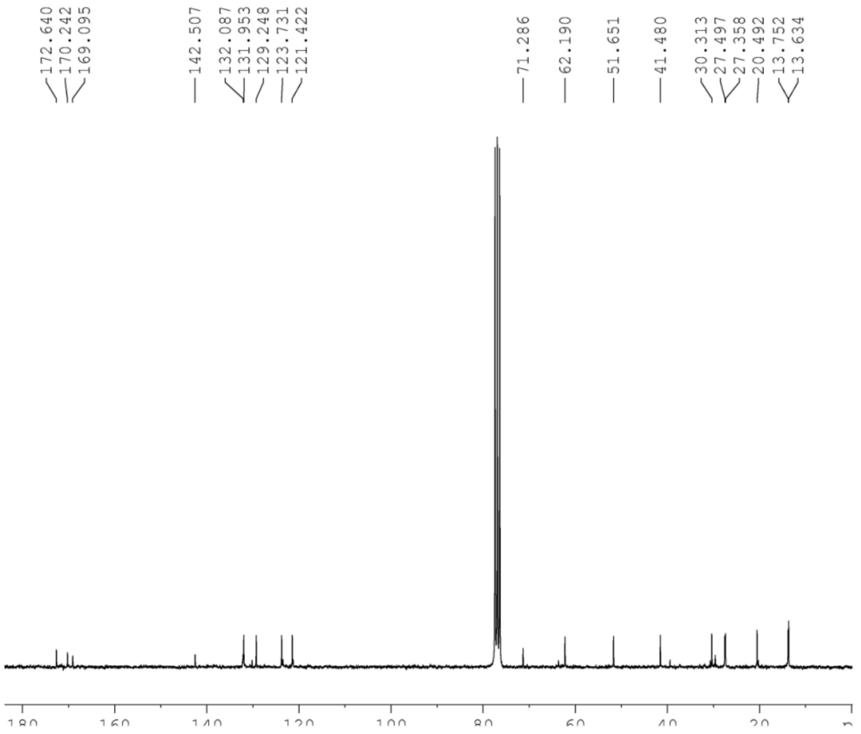

Figure S14. ^{13}C-NMR of compound **6f**.

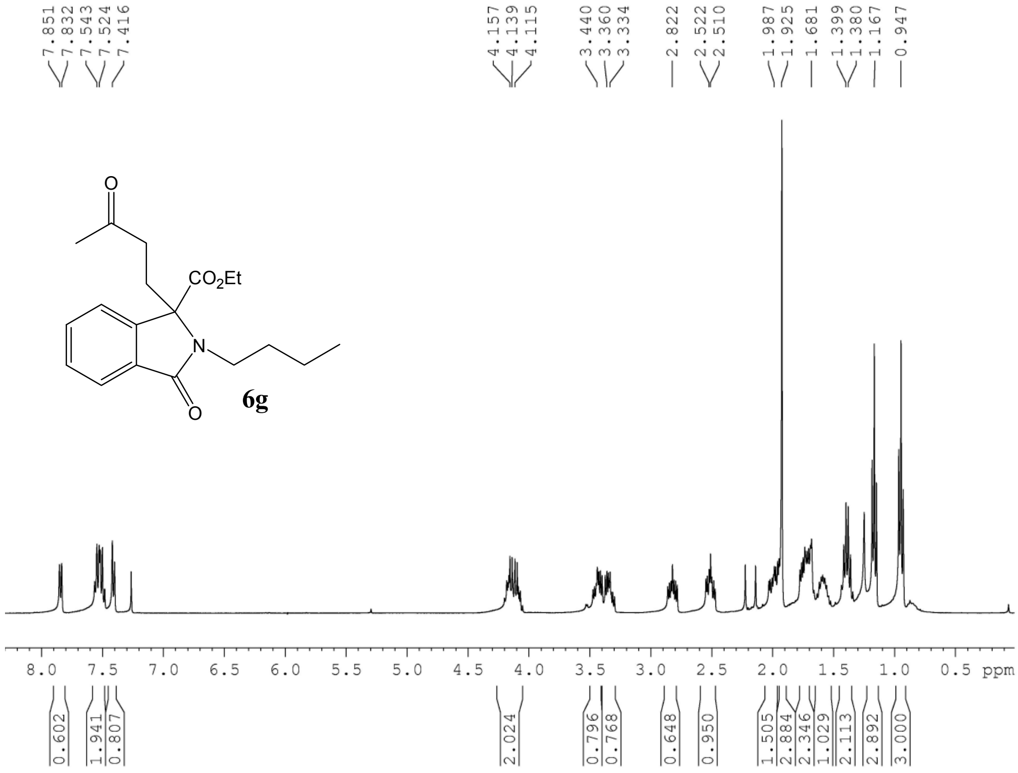

Figure S15. ^{1}H-NMR of compound **6g**.

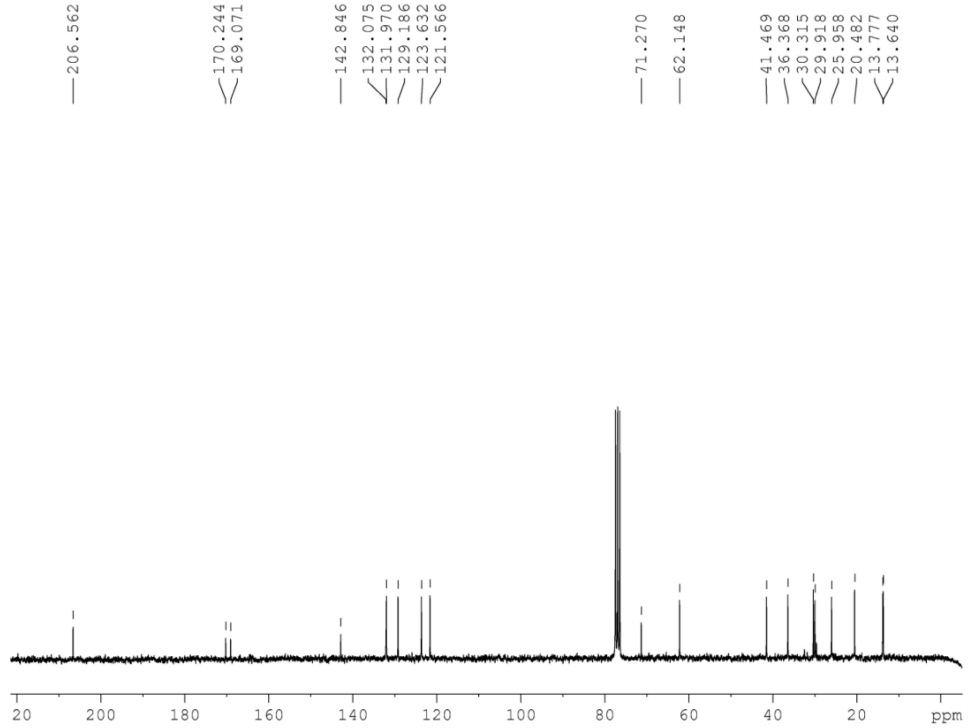

Figure S16. ^{13}C-NMR of compound **6g**.

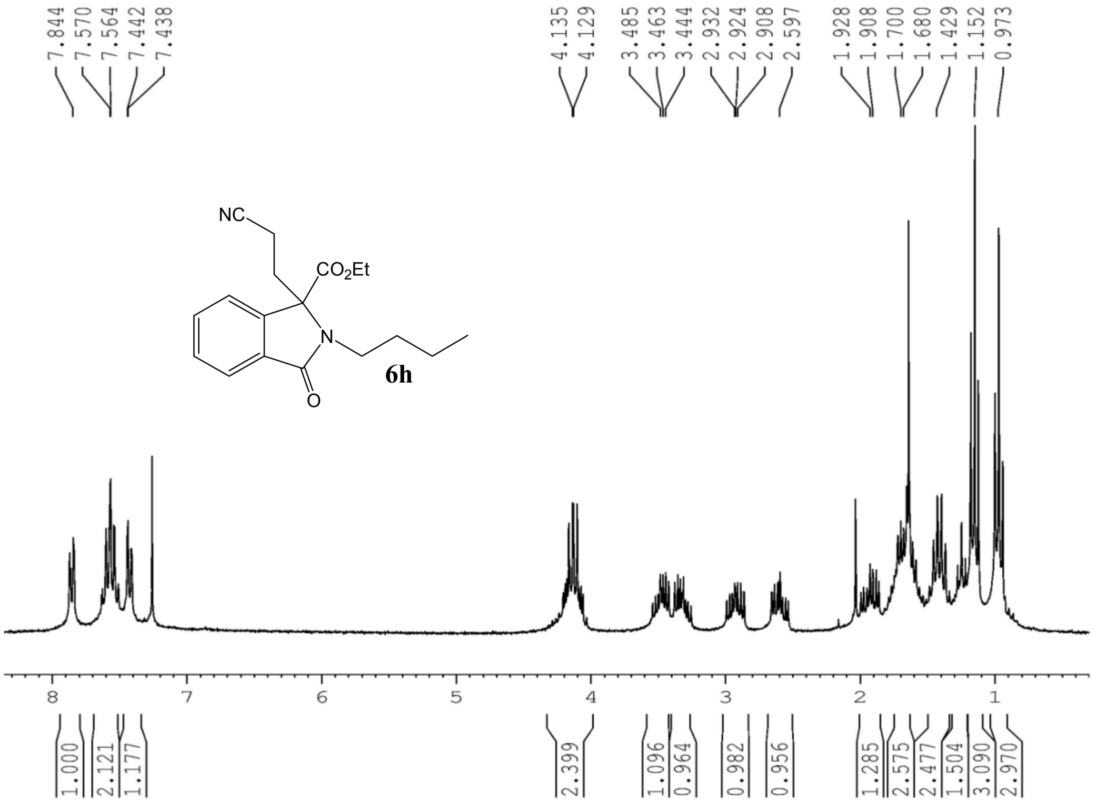

Figure S17. ^{1}H-NMR of compound **6h**.

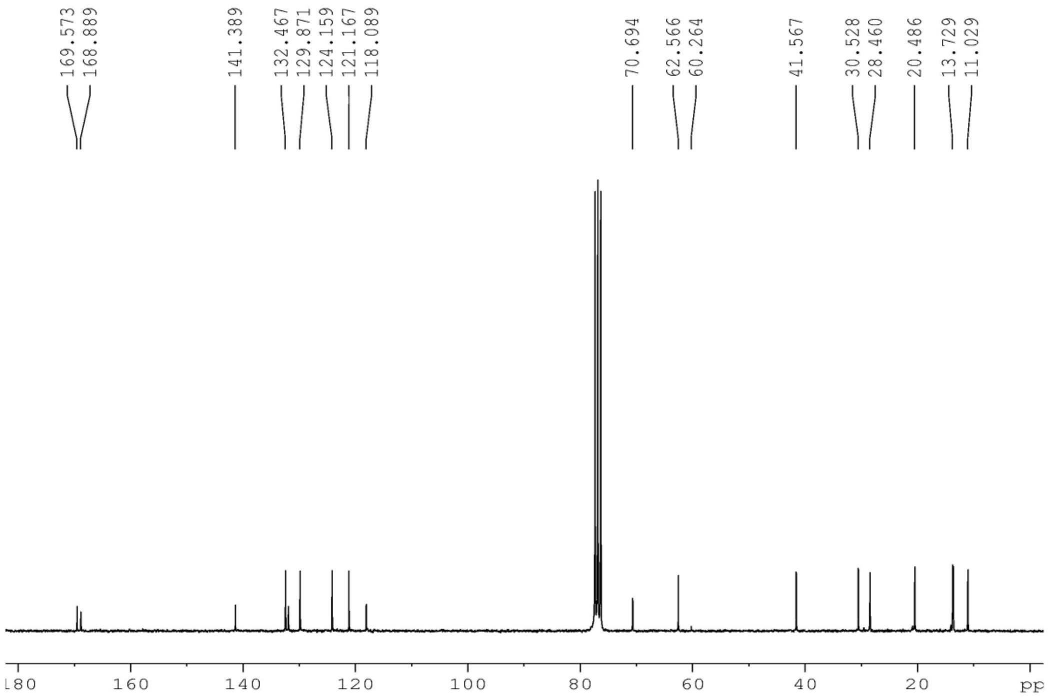

Figure S18. ^{13}C-NMR of compound **6h**.

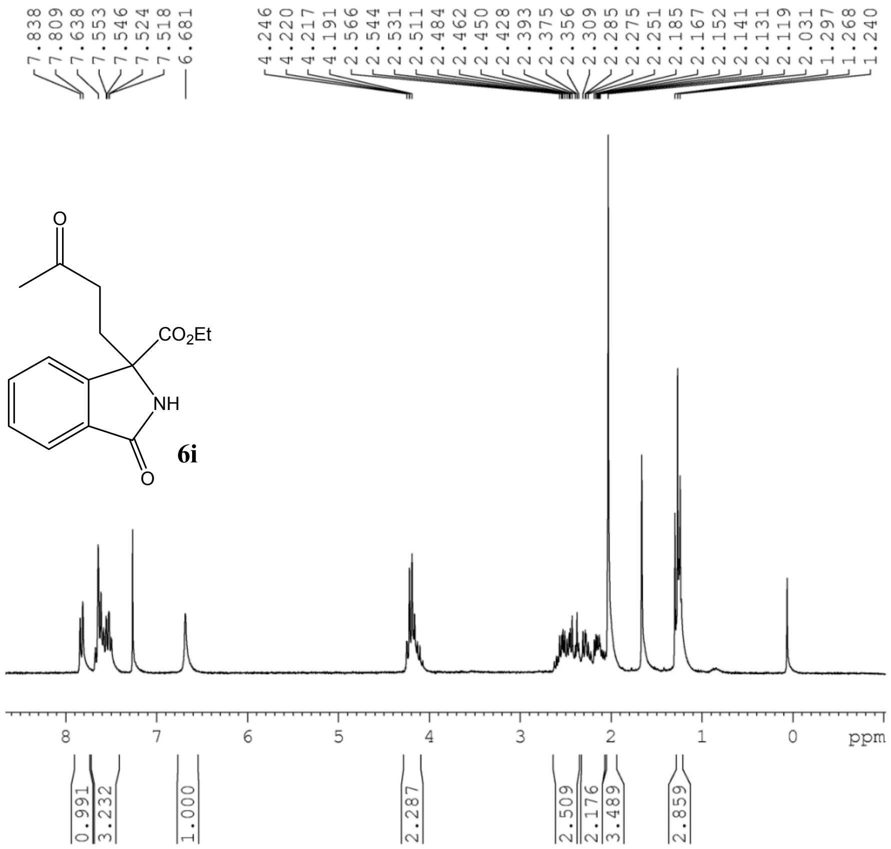

Figure S19. ^{1}H-NMR of compound **6i**.

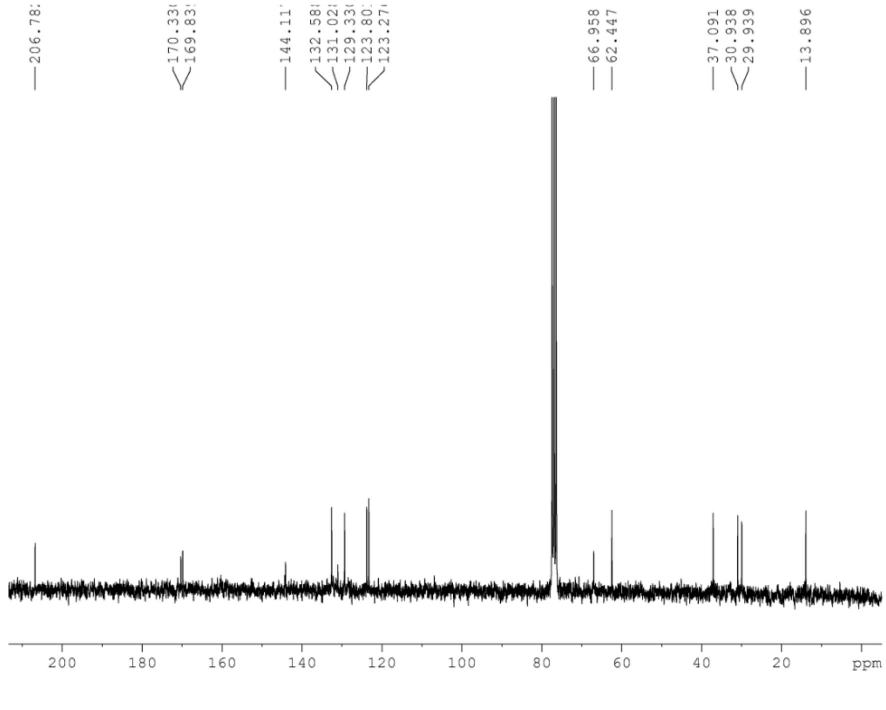

Figure S20. ^{13}C-NMR of compound **6i**.

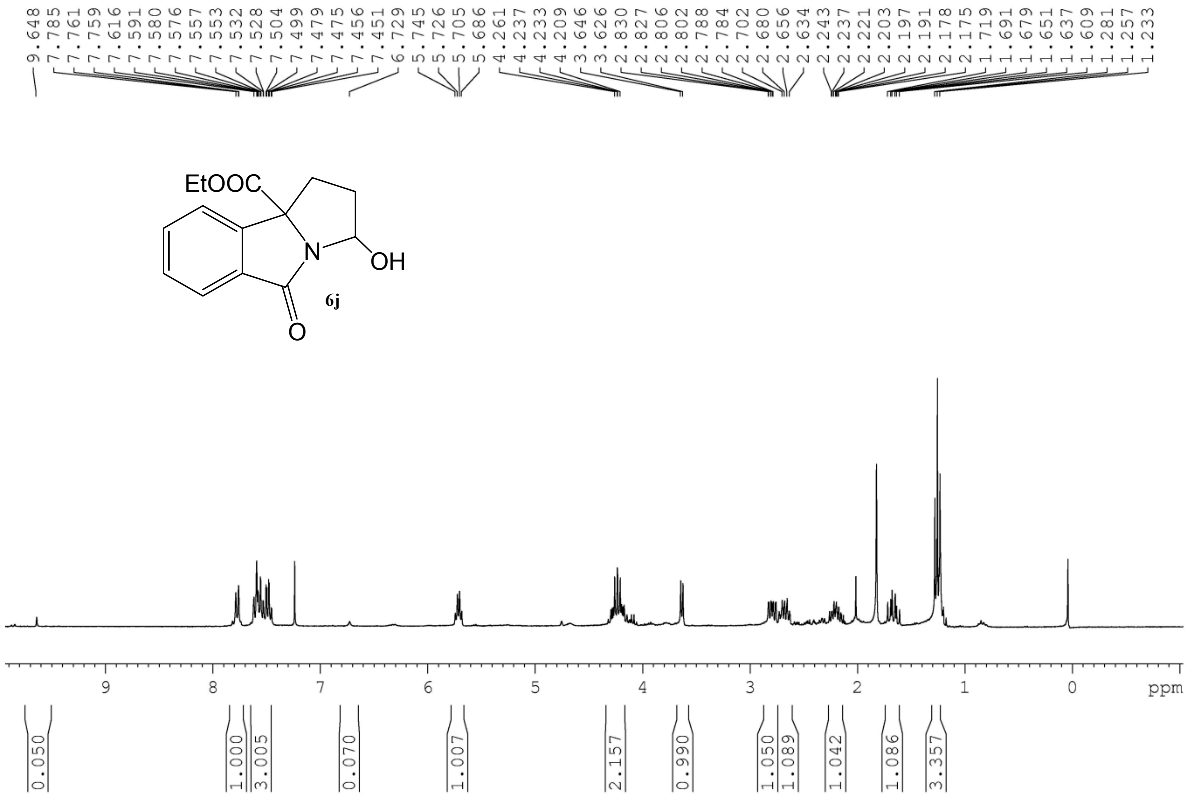

Figure S21. ^{1}H-NMR of compound **6j**.

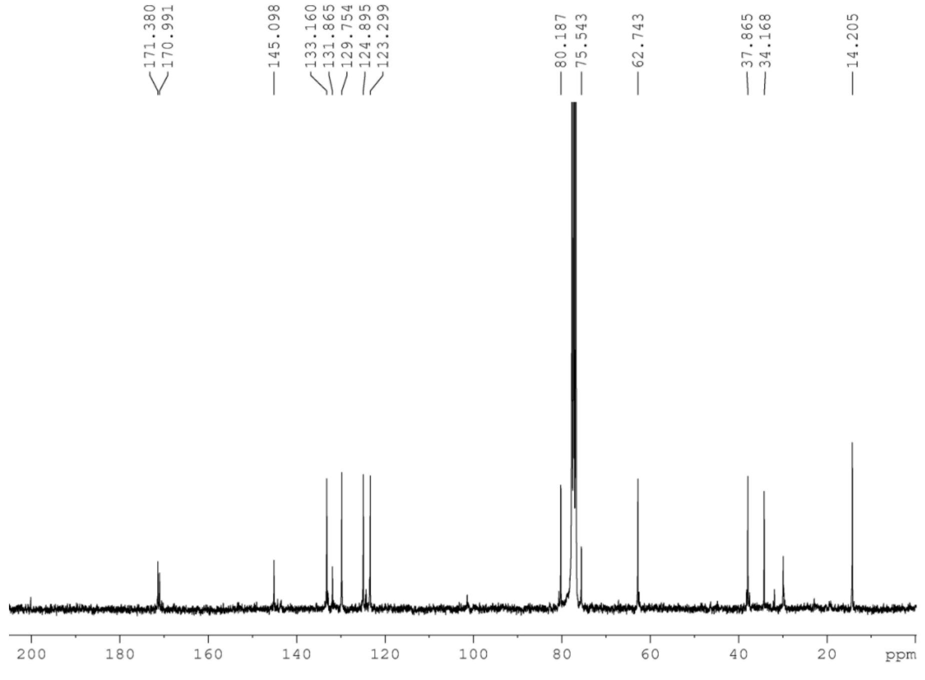

Figure S22. ^{13}C-NMR of compound **6j**.

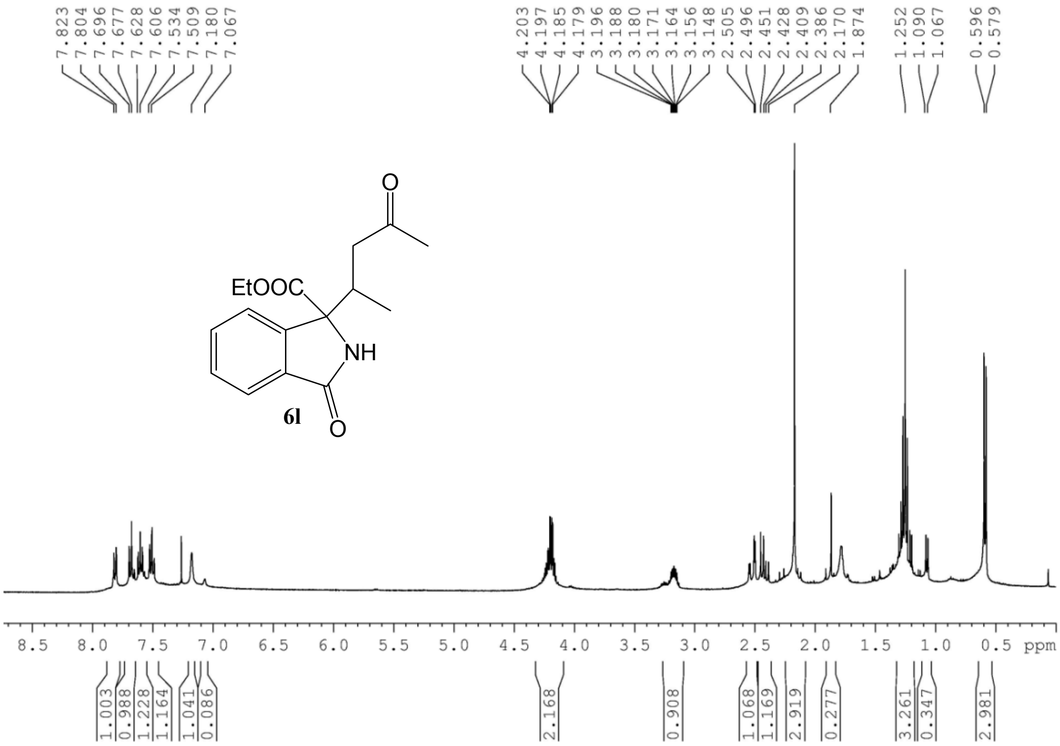

Figure S23. ^{1}H-NMR of compound **6l**.

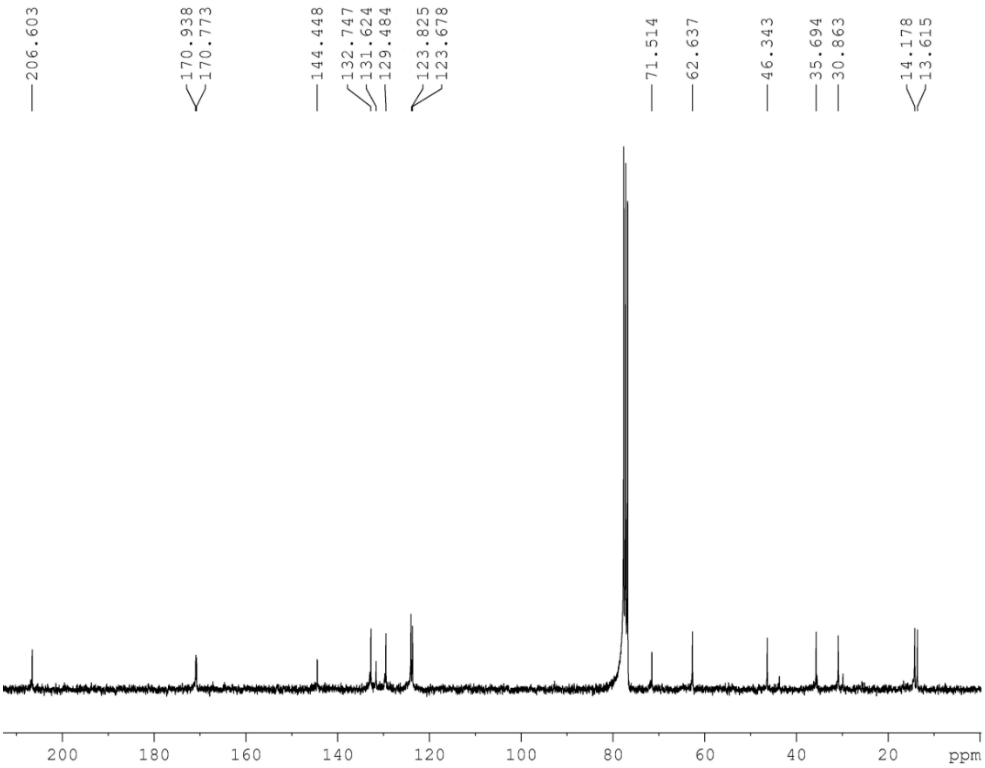

Figure S24. ^{13}C-NMR of compound **6l**.

2. Selected HPLC-Chromatograms(Chiral Stationary Phase)

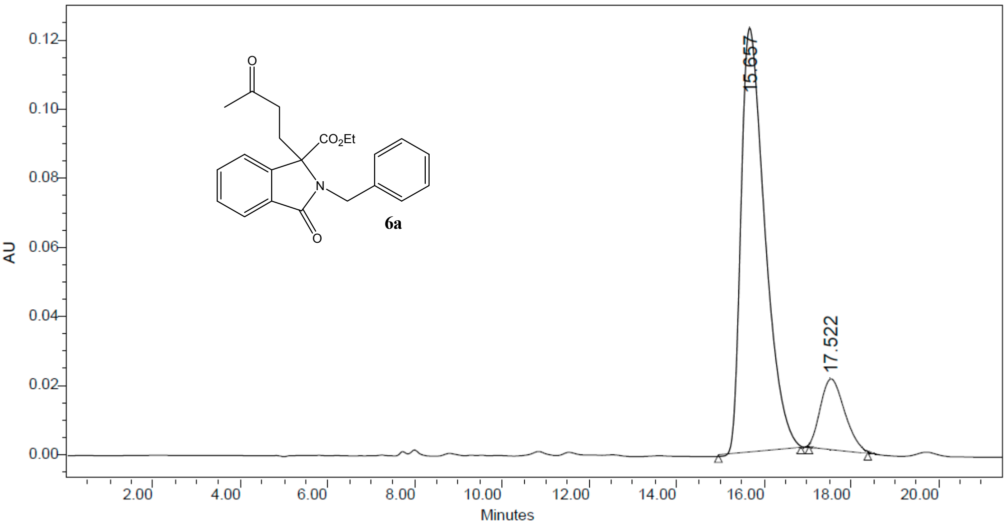

	RT (min)	Area (□V*sec)	% Area	Height (□V)	% Height
1	15.657	4679656	86.62	122939	86.29
2	17.522	722552	13.38	19535	13.71

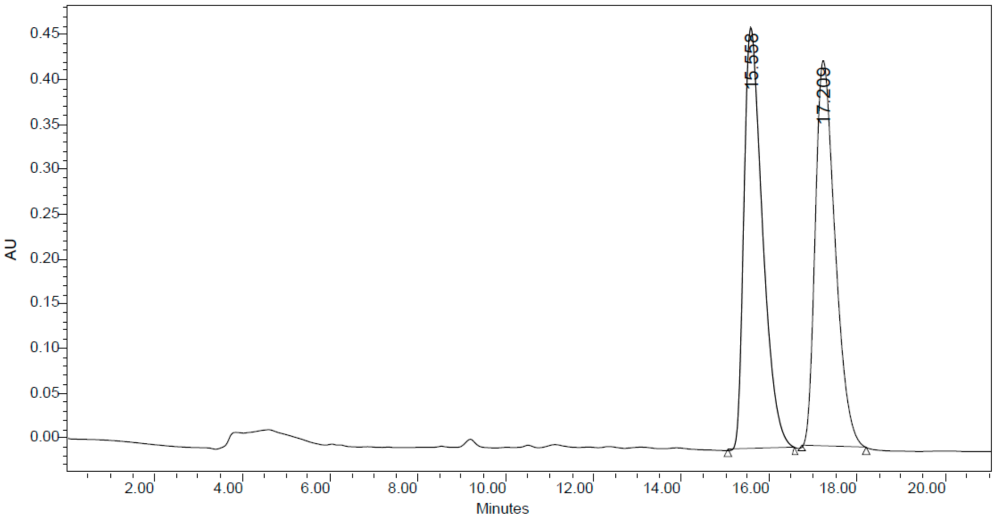

	RT (min)	Area (□V*sec)	% Area	Height (□V)	% Height
1	15.558	13900619	50.73	471059	52.25
2	17.209	13498637	49.27	430555	47.75

Figure S25. HPLC traces of enantioenriched and racemic compound **6a**.

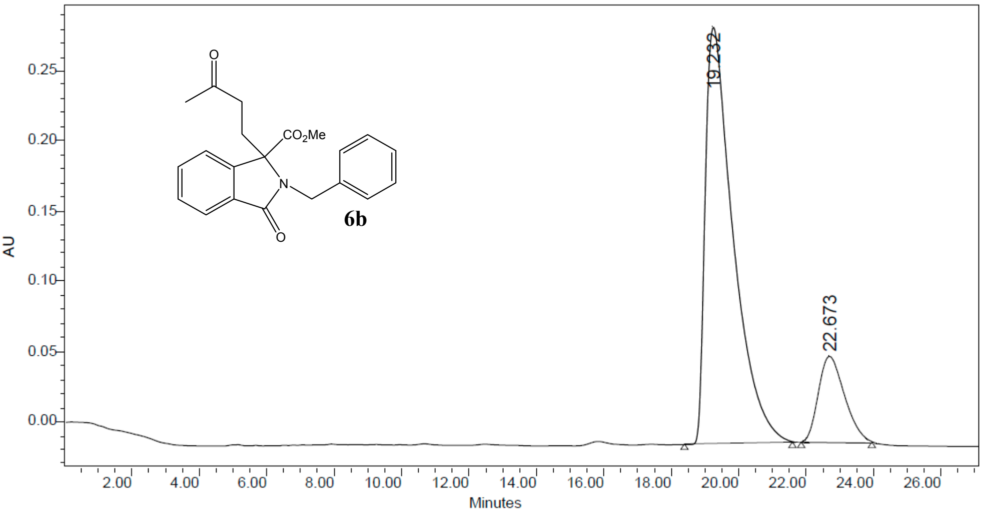

	RT (min)	Area (µV*sec)	% Area	Height (µV)	% Height
1	19.232	17109418	84.88	297081	83.41
2	22.673	3047868	15.12	59079	16.59

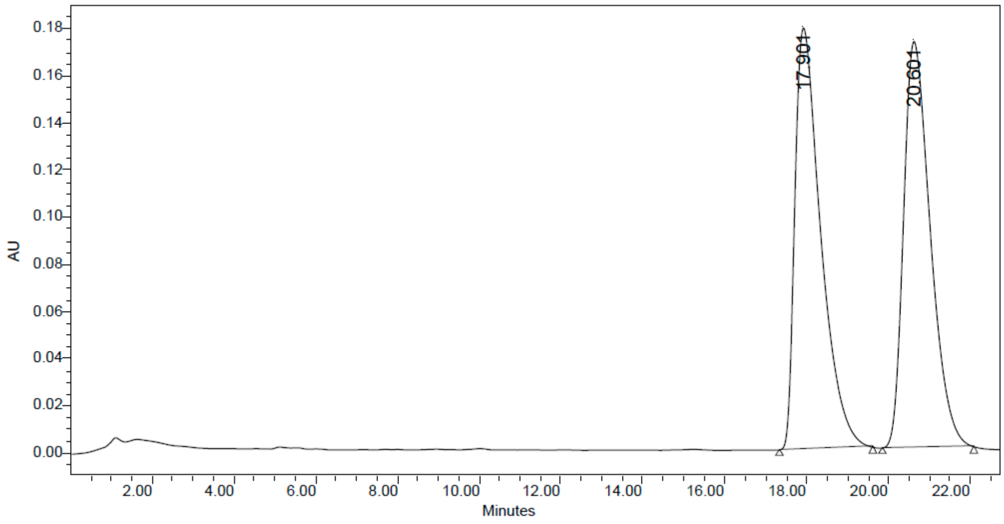

	RT (min)	Area (µV*sec)	% Area	Height (µV)	% Height
1	17.901	7851411	50.07	178739	50.92
2	20.601	7828373	49.93	172305	49.08

Figure S26. HPLC traces of enantioenriched and racemic compound **6b**.

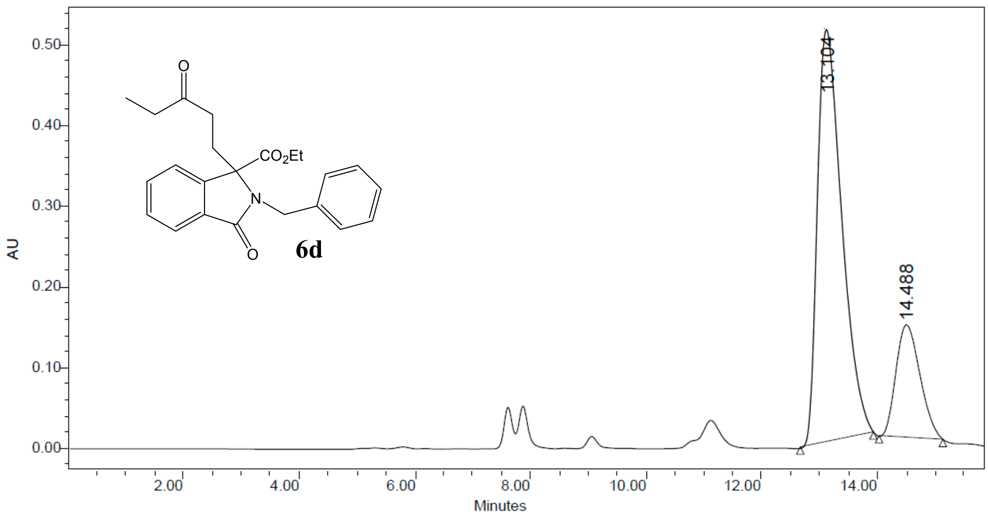

	RT (min)	Area (µV*sec)	% Area	Height (µV)	% Height
1	13.104	15103002	78.77	512601	78.55
2	14.488	4069992	21.23	139998	21.45

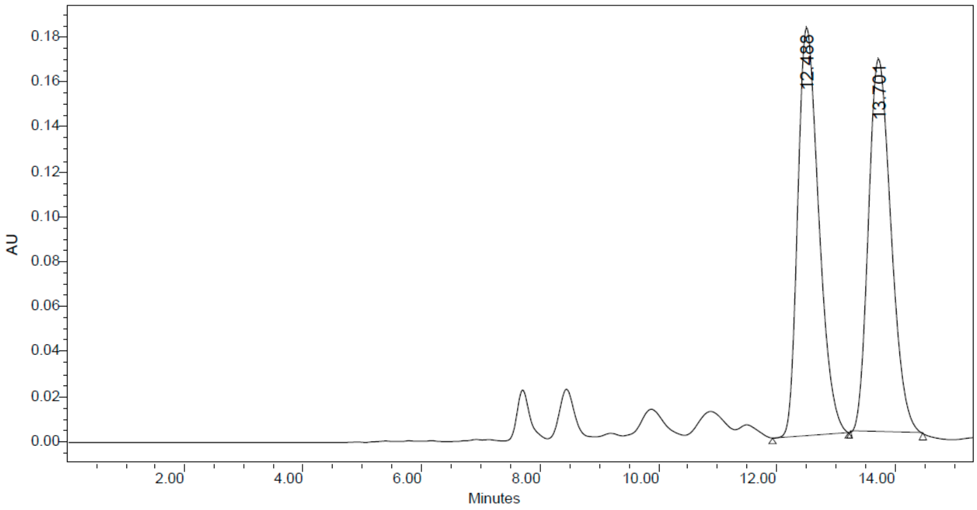

	RT (min)	Area (µV*sec)	% Area	Height (µV)	% Height
1	12.488	4491049	50.22	181728	52.18
2	13.701	4450973	49.78	166535	47.82

Figure S27. HPLC traces of enantioenriched and racemic compound **6d**.

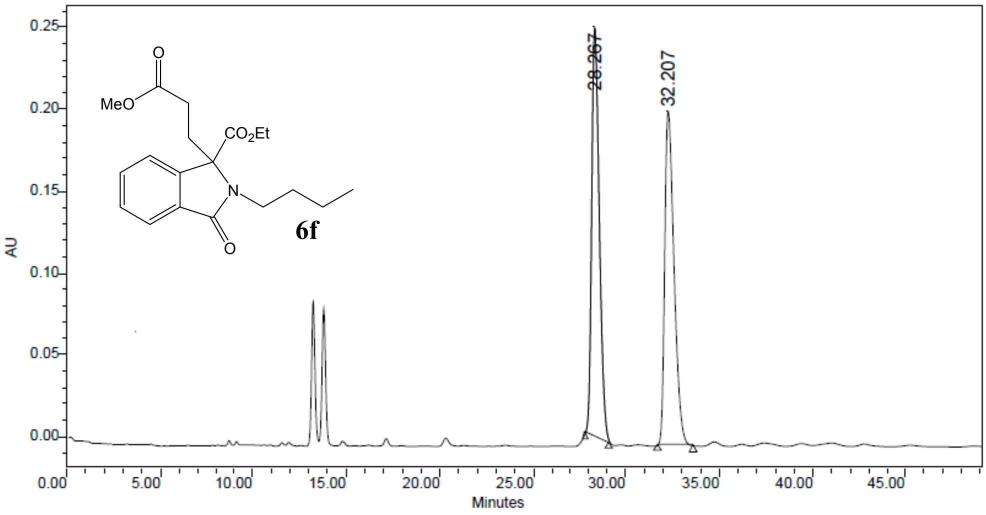

	RT (min)	Area (□V*sec)	% Area	Height (□V)	% Height
1	28.267	7041053	49.92	249223	55.02
2	32.207	7064857	50.08	203764	44.98

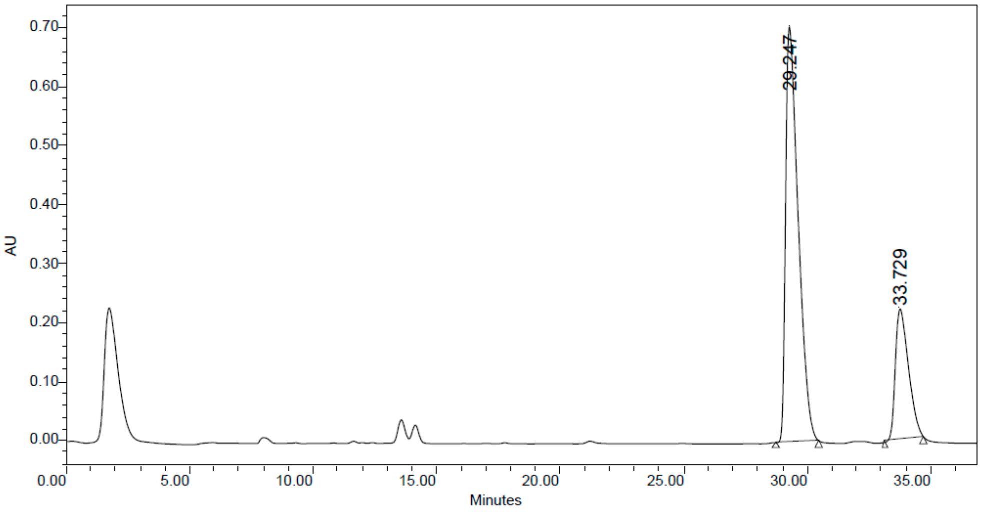

	RT (min)	Area (□V*sec)	% Area	Height (□V)	% Height
1	29.247	25027896	75.04	704285	75.96
2	33.729	8323442	24.96	222852	24.04

Figure S28. HPLC traces of enantioenriched and racemic compound **6f**.

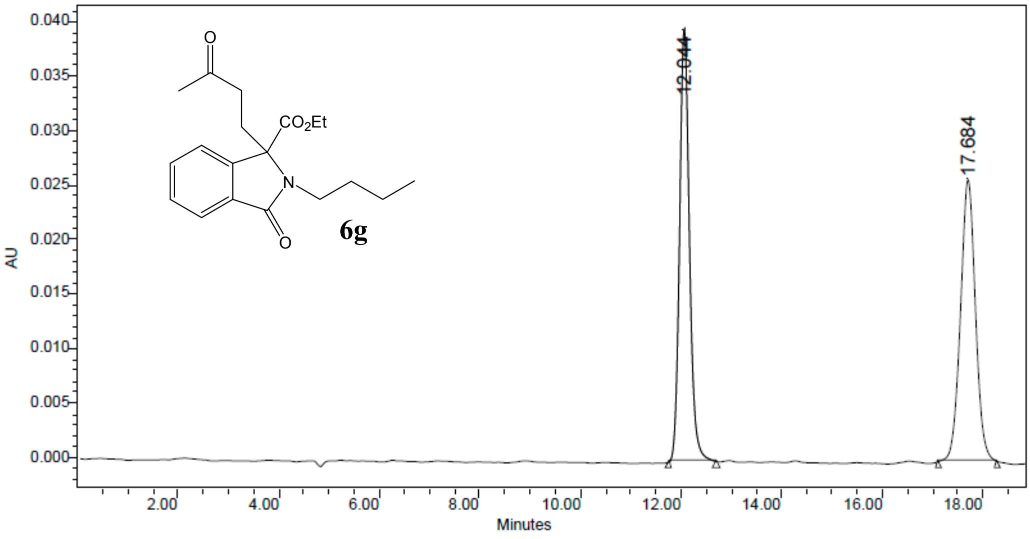

	RT (min)	Area (µV*sec)	% Area	Height (µV)	% Height
1	12.044	540117	50.28	39787	60.62
2	17.684	534089	49.72	25849	39.38

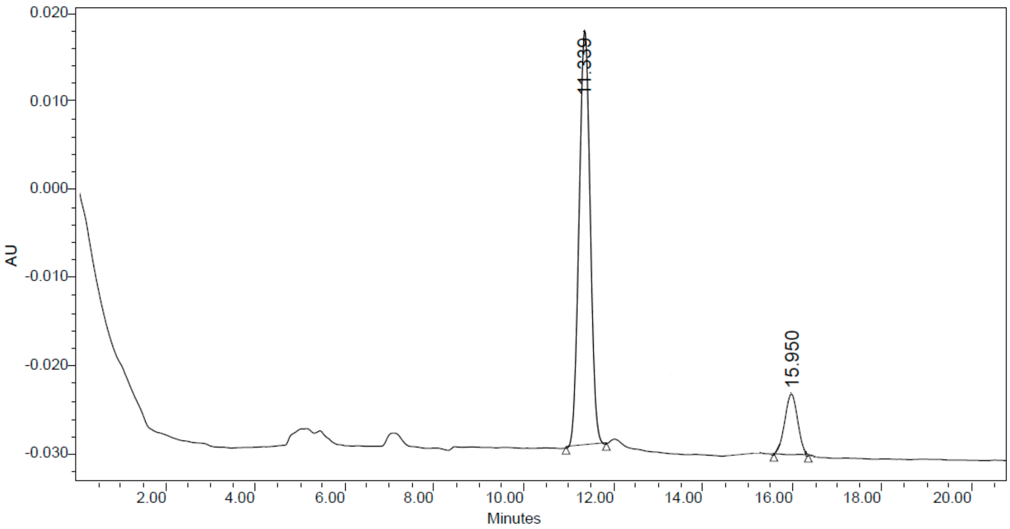

	RT (min)	Area (µV*sec)	% Area	Height (µV)	% Height
1	11.339	812583	88.07	47095	88.43
2	15.950	110026	11.93	6162	11.57

Figure S29. HPLC traces of enantioenriched and racemic compound **6g**.
